# Direct, rapid antimicrobial susceptibility test from positive blood cultures based on microscopic imaging analysis

**DOI:** 10.1038/s41598-017-01278-2

**Published:** 2017-04-25

**Authors:** Jungil Choi, Hyun Yong Jeong, Gi Yoon Lee, Sangkwon Han, Shinhun Han, Bonghwan Jin, Taegeun Lim, Shin Kim, Dong Young Kim, Hee Chan Kim, Eui-Chong Kim, Sang Hoon Song, Taek Soo Kim, Sunghoon Kwon

**Affiliations:** 1 QuantaMatrix Inc., Medical Innovation Center, Seoul National University Hospital, Seoul, 03080 Republic of Korea; 2 Institutes of Entrepreneurial BioConvergence, Seoul National University, Seoul, 08826 Republic of Korea; 3 Program of Nano Science and Technology, Graduate School of Convergence Science and Technology, Seoul National University, Seoul, 08826 Republic of Korea; 4 Department of Electrical and Computer Engineering, Institute of Engineering Research, Seoul National University, Seoul, 08826 Republic of Korea; 5 Institute of Medical and Biological Engineering, Medical Research Center, Seoul National University, Seoul, 03080 Republic of Korea; 6 Department of Biomedical Engineering, Seoul National University College of Medicine, Seoul, 03080 Republic of Korea; 7 Department of Biomedical Engineering, Seoul National University Hospital, Seoul, 03080 Republic of Korea; 8 Department of Laboratory Medicine, Seoul National University Hospital, Seoul, 03080 Republic of Korea; 9 Seoul National University Hospital Biomedical Research Institute, Seoul National University Hospital, Seoul, 03080 Republic of Korea

## Abstract

For the timely treatment of patients with infections in bloodstream and cerebrospinal fluid, a rapid antimicrobial susceptibility test (AST) is urgently needed. Here, we describe a direct and rapid antimicrobial susceptibility testing (dRAST) system, which can determine the antimicrobial susceptibility of bacteria from a positive blood culture bottle (PBCB) in six hours. The positive blood culture sample is directly mixed with agarose and inoculated into a micropatterned plastic microchip with lyophilized antibiotic agents. Using microscopic detection of bacterial colony formation in agarose, the total time to result from a PBCB for dRAST was only six hours for a wide range of bacterial concentrations in PBCBs. The results from the dRAST system were consistent with the results from a standard AST, broth microdilution test. In tests of clinical isolates (n = 206) composed of 16 Gram-negative species and seven Gram-positive species, the dRAST system was accurate compared to the standard broth microdilution test, with rates of 91.11% (2613/2868) categorical agreement, 6.69% (192/2868) minor error, 2.72% (50/1837) major error and 1.45% (13/896) very major error. Thus, the dRAST system can be used to rapidly identify appropriate antimicrobial agents for the treatment of blood stream infection (BSI) and antibiotic-resistant strain infections.

## Introduction

Bloodstream infection (BSI) is a leading cause of morbidity and mortality among hospitalized patients, and approximately 250,000 cases of BSI occur in the U.S. annually^1^. Sepsis, the systemic response to infection including BSI, afflicts approximately 18 million people worldwide each year, and the risk of mortality increases by 9% with every hour that the administration of the correct antibiotic treatment is delayed^2^. Recently, the emergence of antimicrobial resistance became more serious around the world. To prevent the emergence of antimicrobial resistance, the prescription of broad-spectrum antimicrobial drugs is managed by hospital antibiotic stewardship programs^3^. Therefore, to improve the clinical prognosis in patients with antibiotic resistant bacterial infection^4, 5^, fast and accurate determination of the antimicrobial susceptibility of bacteria is mandatory^6–9^.

**Figure 1:**
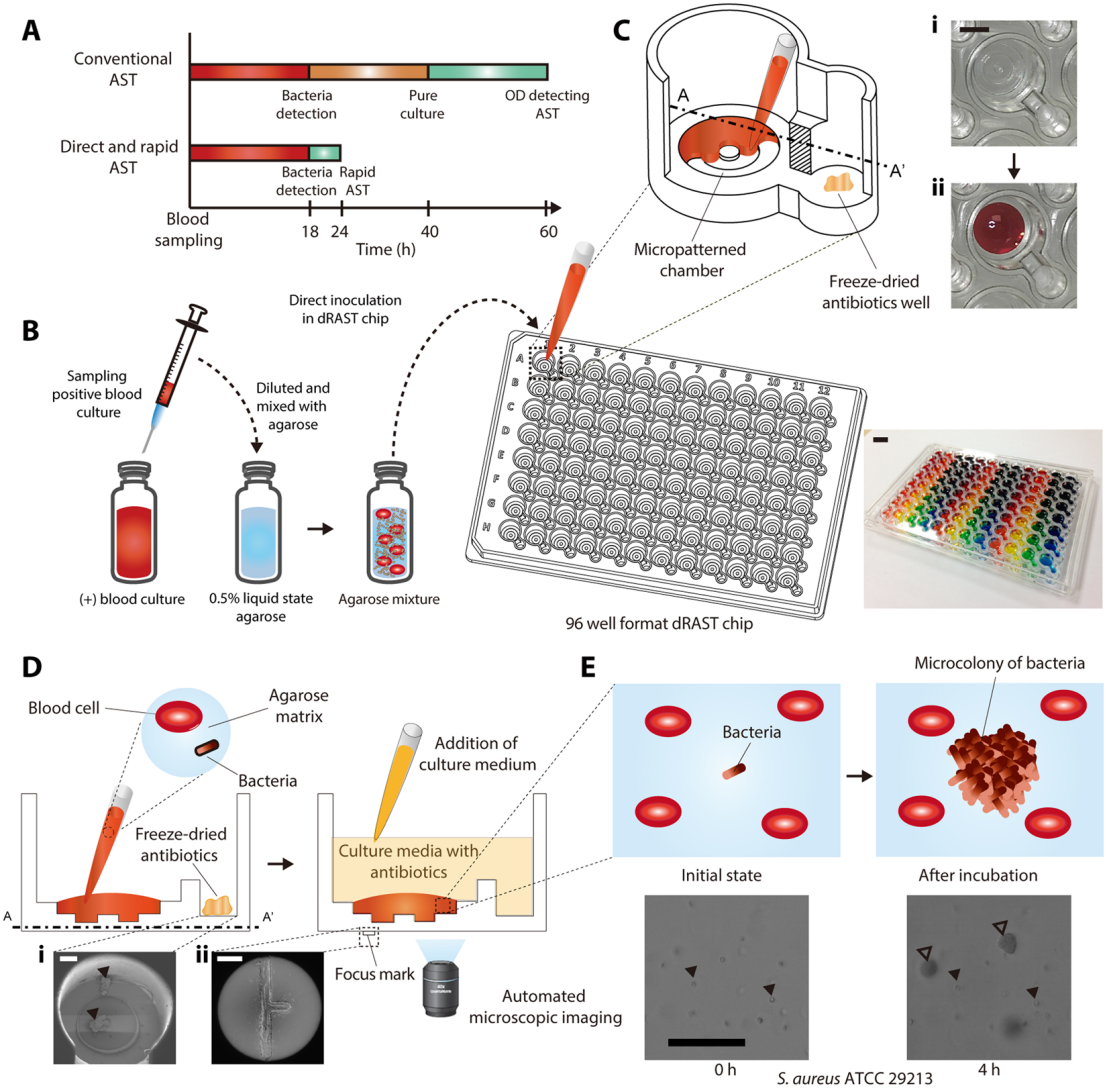
Device for the direct detection of antibiotic resistance from positive blood culture bottles (PBCBs). (**A**) The conventional AST system requires three separate culture processes: blood culture for positive infection detection, subculture for separation of bacteria from blood cells, and AST via optical density measurement; the total time to result is approximately 60 hours. The direct and rapid AST (dRAST) system developed herein does not require a subculture process and enables AST to be performed in six hours. The total time to result of dRAST is less than 24 hours. (**B**) Process of preparing a dRAST chip from a PBCB. A PBCB aliquot was diluted and mixed with liquid-state agarose. The mixture was inoculated into a dRAST chip consisting of 96 test wells containing various antimicrobials at several concentrations. (**C**) Detailed structure of a dRAST chip well. Each test well consists of a micropatterned radial chamber for agarose matrix molding and a satellite well for freeze-dried antibiotics. Inset images show the micropatterned chamber i) before agarose mixture loading and ii) after agarose mixture loading. (**D**) Experimental process for detecting bacteria in blood. A sample from a PBCB is mixed with agarose and loaded into the loading chamber in the chip. Freeze-dried antibiotics are rehydrated by adding culture medium (CAMHB). Automated microscopic imaging is used to detect bacterial growth. Inset scanning electron microscope (SEM) images show (i) freeze-dried antibiotics in the satellite well and (ii) the focus mark on the bottom of the chip for automated imaging. **(E)** In the initial state, bacteria were not detectable using a 20x magnification lens; only blood cells were detectable. After four hours of incubation, in the case of resistance to the antibiotic, a single bacterium divided and formed a microcolony. Filled triangle represents a blood cell. Unfilled triangle represents a bacterial microcolony. The scale bar represents 10 mm in **(B)**, 3 mm in **(C)**, 300 μm in **(D)**, and 100 μm in **(E)**.

However, the total turnaround time (TAT) of current antibiotic resistance detection process for blood samples from BSI patients is longer than three days since it requires three overnight culture steps: blood culture, subculture and antimicrobial susceptibility test (AST) culture (see Fig. 1A). In positive blood culture bottle (PBCB), bacteria are mixed with large number of blood cells and bacterial concentration ranges from 10^7^ to 10^9^ CFU/ml, unpredictably. As conventional AST systems detect changes in bacterial populations by measuring the optical density (OD) of the antibiotic-dosed pathogen culture, they depend very sensitively on the initial inoculum size and initial concentration of bacteria should be strictly controlled. Consequently, the PBCBs cannot be directly used for AST using conventional methods. Therefore, subculture process on agar plate is needed to obtain accurate concentration pure bacterial stock. Using the bacterial stock, over-night AST process is performed.

To reduce the TAT of current antibiotic resistance detection process, various methods have been developed^10–17^. For rapid AST, researchers have developed methods to observe bacterial division at early incubation stages^14–21^. Compared to rapid AST reducing 10–12 hours, removing subculture process can save additional 24 hours. Therefore, Kirby-Bauer disk diffusion method from blood culture inoculum was introduced as direct AST^22–25^. Also, many researchers tried to remove subculture process by separating bacteria from positive blood culture bottles (PBCBs) using centrifugal separation method^26–31^ and blood cell lysis filtration method^32^. The main purpose of these methods is to prepare accurate concentration of bacteria stock for conventional AST. However, these separation processes are time consuming and require multiple manual preparations that are not suitable for clinical application. For rapid AST without subculture process to be utilized in clinical settings, a new AST system handling a wide range of inoculum sizes of bacteria is required.

We have developed a direct AST system that can process a wide dynamic range of inoculum sizes from PBCBs without separation process. By directly processing samples from PBCB without measuring inoculum size, we demonstrated TAT less than 30 hours without sacrificing accuracy (see Fig. 1A). Specifically, we detect the antibiotic resistance of bacteria using microscopic imaging and micropatterned plastic microchips instead of OD measurement. For the interpretation AST from dRAST, bacterial identification is necessary. Therefore, dRAST was simultaneously performed with the direct ID system, MALDI Biotyper and Sepsityper kit from Bruker (Billerica, MA, United States). By satisfying the recommended performance criteria of the United States (U.S.) Food and Drug Administration (FDA), this study demonstrated the feasibility of using the dRAST system in clinical settings as a rapid AST for positive blood culture specimens. The direct use of PBCBs in this rapid AST system reduced the turnaround time to appropriate antimicrobial treatment by two days compared to conventional systems.

## Methods

### Fabrication of the dRAST chip

The dRAST chip (96-well format) was designed using 3D CAD design software (SolidWorks v2014, Dassault Systèmes SolidWorks Corp., Vélizy-Villacoublay, France) and was fabricated by an injection molding (NT2–120, Woojin Plaimm, Republic of Korea) of polystyrene (K-RESIN, Chevron Phillips Chemical, TX, United States). Prior to AST, a one-minute air plasma treatment (CUTE-MP, Femto Science, Republic of Korea) was used for hydrophilic treatment of the chip.

### Quality control strains

The four Clinical and Laboratory Standards Institute (CLSI) standard strains (*E. coli* ATCC 25922, *S. aureus* ATCC 29213, *P. aeruginosa* ATCC 27853, and *E. faecalis* ATCC 29212) were purchased from MicroBioLogics, Inc. (MN, United States). Stock solutions were made using 25% glycerol (Sigma-Aldrich, MO, United States) and were stored at −70 °C. For the test, the stock solution was inoculated into LB agar and incubated at 37 °C overnight.

### Positive blood culture bottles

The clinical evaluation portion of this study was conducted during working hours from June 2015 to December 2015 at Seoul National University Hospital. During the study period, BACTEC Plus Aerobic/F and Anaerobic/F culture bottles that were flagged as positive for microbial growth by a BACTEC FX automated incubation system (Becton Dickinson Company, NJ, United States) and BacT/Alert FA Plus and SN bottles in a BacT/Alert^®^ 3D system (bioMerieux Inc., Marcy l'Étoile, France) were subjected to parallel testing using dRAST and the conventional procedure with the manual broth dilution test. We used the PBCBs flagged as positive during the over-night culture period until 9 AM. All samples from which a single bacterial species determined by Gram staining, recovered by conventional processing (monobacterial samples) were included in a comparative analysis of the identification results. PBCBs were supplied by Seoul National University Hospital (SNUH). Subcultures of the clinical strains from PBCBs were inoculated on Luria-Bertani (LB) agar plates (BD Biosciences, CA, United States) and incubated for 20 to 24 hours. After incubation, several colonies were used to prepare bacterial stocks at a concentration of 1.5 × 10^8^ CFU/ml. Bacterial identification was performed according to the protocols of the hospital where the strain originated: Gram-negative strains were identified by VITEK ID-GN cards in Vitek2 Systems (bioMerieux Inc., Marcy l'Étoile, France) and Gram-positive strains by Pos Breakpoint Combo Panel Type 28 in MicroScan (Beckman Coulter Inc., CA, United States) at SNUH.

### Number of bacteria in PBCBs

The number of bacteria in a PBCB is important for determining the dilution factor. Conventional blood culture bottles detect the CO_2_ generated from bacterial metabolism, which does not precisely represent the number of bacteria in the culture bottle. To determine the number of bacteria in a PBCB, we performed colony forming unit (CFU) detection. We diluted PBCB sample with cation-adjusted Mueller-Hinton broth (CAMHB, BD Biosciences, CA, United States) by 10^6^ fold and inoculated 100 μl onto an agar plate. After counting the colonies formed, we estimated the number of bacteria (see Supplementary Fig. S1).

### Procedure of dRAST

Upon a positive signal from the BACTEC FX (Becton Dickinson Company, NJ, United States) or BacT/ALERT^®^ (bioMerieux Inc., Marcy l'Étoile, France) 3D automated blood culture instrument, a sample was collected from the BACTEC or BacT/ALERT^®^ blood culture bottle with a 1 ml syringe. After Gram staining, polymicrobial and yeast samples were excluded for further test. Substances in the bottles such as antimicrobial agents or antimicrobial absorption materials could affect the AST results. To achieve the optimum inoculation concentration and to eliminate the effects of substances in the bottles, a 10 μl sample was withdrawn from the bottle and diluted 100-fold with 990 μl of CAMHB (see Supplementary Fig. S2C). Then, 300 μl of the diluted sample was mixed with 900 μl of liquid-state 0.5% agarose at 37–40 °C at a 1:3 volume ratio. Next, 10 μl of the mixture was inoculated in the radial-shaped chamber of a 96-well format dRAST chip (see Fig. 1B). Due to the capillary effect, the micro-patterned radial shape of the well helped the agarose mixture spread and form a disk matrix in the entire well (see Fig. 1C,Ci and Cii). This micro-patterned radial shape of the well eliminated occasional diffusion limit occurred in previous researches with firm stabilization of agarose mixture to the bottom side of dRAST chip^19, 20^. After solidification of the agarose mixture at room temperature, 100 μl of culture medium was loaded into the satellite well to rehydrate the freeze-dried antibiotics (see Fig. 1D and Di). The culture medium with antibiotics was diffused into the agarose matrix. The imaging plane in the z-axis was 150 μm from the interface between the agarose and antibiotics. The thickness of the agarose matrix was 300 μm, and diffusion was sufficient in all regions of the agarose^20^. The total hands-on time for the preparation of the 96-well dRAST chip was less than five minutes. The chip was prepared and incubated in a 37 °C chamber. A focus mark was imprinted on the bottom of the chip for automatic tracking of the image region in the chip (see Fig. 1Dii). Using the focus mark, the same location in the agarose was imaged every two hours using a time-lapse method. The field of view of one image was 1126.4 × 594 μm. At the starting point, only hemocytes were detectable in the image. During incubation, the bacteria in the agarose formed a three-dimensional colony, which was detected after four hours (see Fig. 1E). Thus, we considered microcolony formation to indicate a case of growth due to the resistance of the bacteria to a certain antimicrobial. Cases with no microcolony formation were considered indicative that the bacterial strain was susceptible to the antimicrobial.

### dRAST test with spike-in sample

Four standard CLSI strains– *E. coli* ATCC 25922, *S. aureus* ATCC 29213, *P. aeruginosa* ATCC 27853, and *E. faecalis* ATCC 29212 - were cultured on LB agar plates. Then, 100 μl of stock solution containing approximately 10 CFU of bacteria was inoculated into blood culture bottles. To each bottle, 8 ml of fresh human blood from healthy volunteers was added to mimic blood culture bottles. The spiked bottles were incubated in BACTEC and BacT/Alert automated blood culture instruments. After the positive detection of bacteria, sampling was performed for dRAST. Briefly, 10 μl of sample was diluted with 990 μl of CAMHB, and 300 μl of the diluted sample was mixed with 900 μl of 0.5% agarose. Then, 10 μl of the mixture was inoculated into a micropatterned chamber in a dRAST chip. Time-lapse imaging was performed at 0, 2, 4, and 6 hours.

### dRAST with various inoculum sizes

Bacterial stocks were prepared from bacterial colonies cultured on LB agar plates. The cell density was adjusted based on UV spectrophotometric measurements (DensiCHEK™, bioMerieux Inc., Marcy l'Étoile, France). For the accurate calculation of inoculum size, the bacterial stock was cultured on an LB agar plate to determine the colony-forming units.

### Direct identification of bacteria from positive blood culture bottles

A sample from a PBCB, the BACTEC Plus Aerobic/F, Lytic/10 and Anaerobic/F (Becton Dickinson Company, NJ, United States) or BacT/ALERT FA Plus and SN (bioMerieux Inc., Marcy l'Étoile, France), was processed using the Sepsityper kit (Billerica, MA, United States) prior to analysis using the Bruker MALDI-TOF Biotyper system (3.1 software). Briefly, 1.0 ml of sample from a PBCB was transferred to a 1.5 ml centrifuge tube (provided). A 200 μl aliquot of lysis buffer (provided) was added to the blood specimen, and the mixture was vortexed for 10 s prior to centrifugation (13,000 rpm, 2 min). Following centrifugation, the supernatant was removed, and the bacterial pellet was resuspended in 1.0 ml of wash buffer (provided), vortexed, and centrifuged (13,000 rpm, 1 min). The supernatant was discarded, and the pellet was resuspended in 300 μl of deionized water. Then, 900 μl of 100% ethanol was added, and the mixture was vortexed and centrifuged at 13,000 rpm for 1 min. The ethanol was discarded, and the sample was again centrifuged at 13,000 rpm for 1 min. The pellet was allowed to dry completely. When dry, 70% formic acid (Sigma-Aldrich, MO, United States) at the same volume as the pellet (~10 μl) was added and mixed. The same volume (~10 μl) of acetonitrile (Sigma-Aldrich, MO, United States) was added, and the pellet was resuspended. The suspension was centrifuged a final time (13,000 rpm, 1 min), and 1 μl of the resulting supernatant was analyzed using MALDI-TOF MS.

### Clinical Study of dRAST with MALDI-TOF MS

To demonstrate the performance of integrated direct ID using MALDI-TOF MS and direct AST using the dRAST system, we utilized a Sepsityper from Bruker for direct ID of bacteria from PBCB. From the flagged PBCB sample, direct ID was performed with the Bruker Biotyper while direct and rapid AST was simultaneously performed with the dRAST system. Using the ID information from MALDI-TOF, we interpreted MIC results provided by the dRAST system (see Supplementary Table S1). This study was approved by the SNUH IRB (# 1312-107-546) and was conducted at SNUH from 6/23/15 to 12/16/15. All methods related with using PBCBs and data from Vitek2 and MicroScan were carried out in accordance with relevant guidelines and regulations from SNUH IRB. All experimental protocols of performing ID and AST were approved SNUH IRB. From the regulation from SNUH IRB, informed consent from subjects was exempted because the experiment used only the information of bacteria from the PBCBs and not human related information. We performed the clinical testing as part of a prospective study. The sample included duplicate blood culture bottles, which were originated from the same patient, only incubated in separate blood culture bottles due to different blood extraction sites (Data File S1). After Gram-staining, only monomicrobial isolates from positive blood bottles were studied. After direct ID using MALDI-TOF, samples with low MALDI scores (<1.7) were excluded due to low credibility of data. Also, streptococcal strains were excluded because they need lysed horse blood (2.5% to 5% v/v) in culture media. Samples with unreliable growth detection in the control well were also excluded (see Supplementary Table S2)^33, 34^.

### Statistical analysis

The concordance of susceptibility results was determined using the categorical agreement and discrepancy rates for the detection of antimicrobial susceptibility with 95% confidence intervals (CI). The 95% CIs for the proportions of categorical agreement between the dRAST and gold standard BMD method, including the mE, ME and VME, were also calculated. All the statistical analyses were performed using SAS (Version 9.3, SAS Institute, NC, United States).

## Results

### Direct detection of antibiotic resistance from positive blood culture bottles

The raw sample contained an average of 10^9^ CFU/ml of bacterial cells mixed with hemocytes, and the microcolonies were too small for their formation to be detected (see Supplementary Fig. S2A,B). To validate the accuracy of the dRAST system, we performed direct AST using spiked samples of four standard Clinical and Laboratory Standards Institute (CLSI) strains – *E. coli* ATCC 25922, *S. aureus* ATCC 29213, *P. aeruginosa* ATCC 27853, and *E. faecalis* ATCC 29212 – in blood culture bottles containing fresh human blood. When the PBCB sample was mixed with agarose, only hemocytes of size 7–15 μm were initially detectable (see Fig. 1E). After incubation, single bacterial cells in the agarose had divided, and each had formed a microcolony larger than 20 µm, which was detectable (see Fig. 1E). Using this scheme, we could determine antibiotic resistance directly from PBCBs. The MIC was derived using microcolony detection from time-lapse images and was compared with the MIC value from the BMD test, the standard AST method (see Supplementary Table S5). The MIC values from dRAST were consistent with the values from BMD and were within the CLSI quality control (QC) ranges. This supports that the results from dRAST chip using colony detection are coincident with standard test. Thus, results from dRAST are feasible to determine the AST results of bacteria with antibiotics. Therefore, the detection of bacterial microcolonies in an agarose matrix was validated as a direct and rapid AST method for PBCB samples.

### Consistent AST results from wide range of inoculum sizes

**Figure 2:**
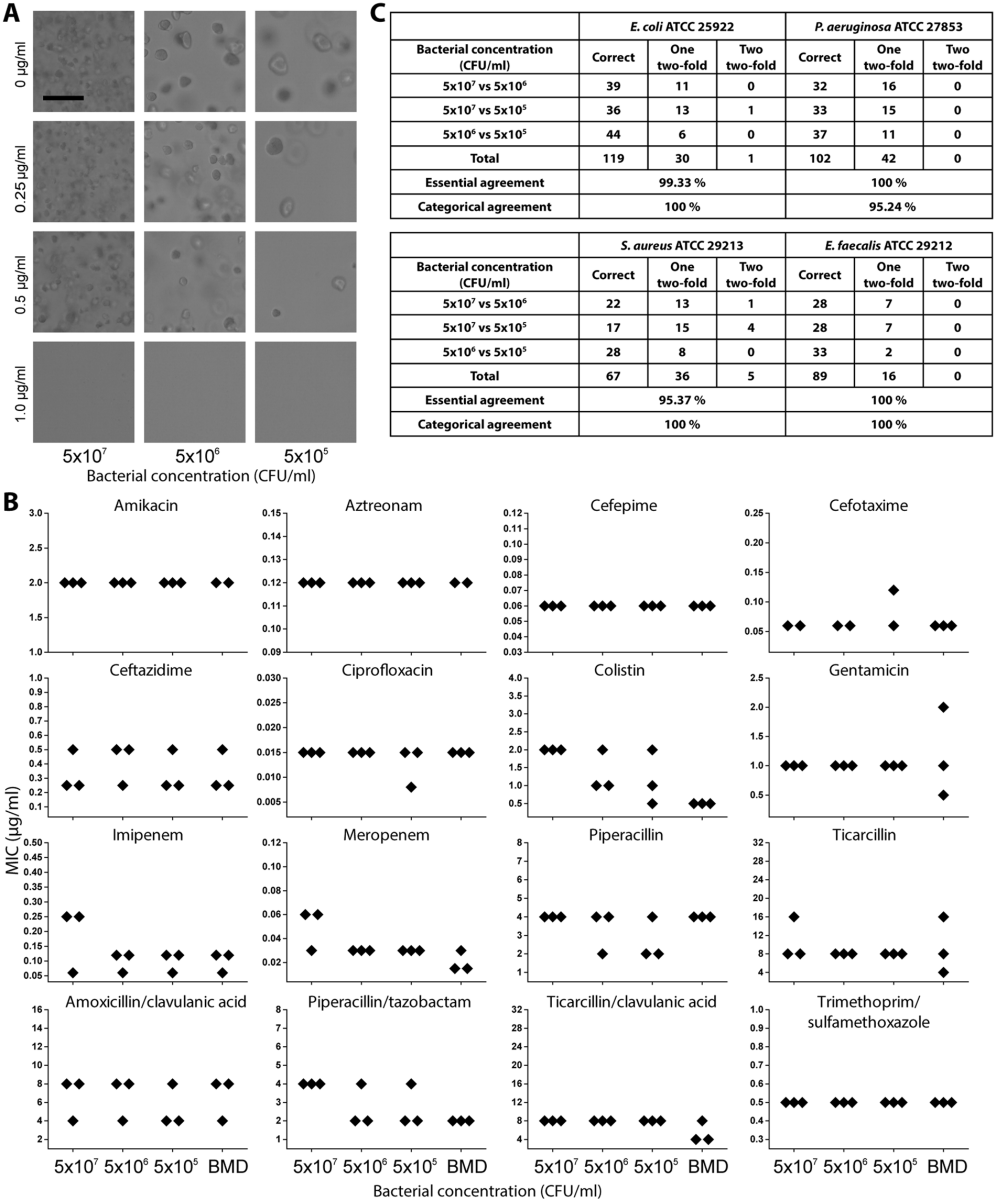
Consistent AST results from a wide range of inoculum sizes. (**A**) Colony formation of bacteria at different inoculum sizes. In all inoculum sizes from 5.0 × 10^7^ to 5.0 × 10^5^ CFU/ml, there was microcolony formation at 0, 0.25 and 0.5 μg/ml gentamicin. At 1 μg/ml gentamicin, there was no colony formation at any inoculum size. The scale bar represents 100 μm. (**B**) MIC values from different *E. coli* ATCC 25922 inoculum sizes of 5.0 × 10^7^, 5.0 × 10^6^ and 5.0 × 10^5^ CFU/ml incubated with 17 clinically important antimicrobials and analyzed using dRAST and broth microdilution test. For all inoculum sizes, the MIC values were in the middle of the CLSI quality control ranges. **(C)** Summary of a comparison of AST results from various inoculum sizes of *E. coli* ATCC 25922, *P. aeruginosa* ATCC 27853, *S. aureus* ATCC 29213 and *E. faecalis* ATCC 29212. “Correct” means a case with identical MIC values from different inoculum sizes. “One two-fold” and “Two two-fold” denote cases with MIC values from different inoculum sizes that were different by two-fold and four-fold, respectively. “Essential agreement” refers to the proportion of cases that were correct or had one two-fold difference. The essential agreement rates were 99.33% in *E. coli* ATCC 25922, 100% in *P. aeruginosa* ATCC 27853, 95.37% in *S. aureus* ATCC 29213 and 100% in *E. faecalis* ATCC 29212.

Conventional AST systems require a precise bacterial inoculum size in the test well for accurate AST results^35^. Therefore, another obstacle to direct AST from PBCBs is that the bacterial concentration has a wide range and cannot be measured by the culture detection instrument. We measured the bacterial concentrations of PBCBs by observing colony formation on Luria-Bertani (LB) agar plates and found that the concentrations ranged widely, from 5.6 × 10^7^ to 2.6 × 10^9^ CFU/ml (see Supplementary Fig. S1). In the dRAST system, we diluted the PBCB sample at a 1:100 volume ratio, resulting in bacterial concentrations from approximately 5.0 × 10^5^ to 5.0 × 10^7^ CFU/ml. The dRAST system should produce consistent AST results from this inoculum size range. To validate that the dRAST system could perform reliably at this wide range of inoculum sizes, we tested four standard strains, *E. coli* ATCC 25922, *P. aeruginosa* ATCC 27853, *S. aureus* ATCC 29213 and *E. faecalis* ATCC 29212, at inoculum sizes of 5.0 × 10^5^, 5.0 × 10^6^ and 5.0 × 10^7^ CFU/ml. The validation concentrations were determined based on the bacterial concentrations measured in the PBCBs. *E. coli* ATCC 25922 at three inoculum sizes – 5.0 × 10^5^, 5.0 × 10^6^ and 5.0 × 10^7^ CFU/ml – was imaged after four hours of incubation with or without gentamicin (see Fig. 2A). In the absence of antimicrobials, microcolonies formed at all the inoculum sizes. There were approximately 10~1,000 cells in the whole image view (1126.4 × 594 μm) (see Supplementary Table S6). The size of the colony varied according to the inoculum size (see Fig. 2A). We assumed that this behavior was due to quorum sensing in the bacteria; at high densities, bacteria produce and release chemical signal molecules to control cell density^36, 37^. Inoculum size showed an inverse relationship with colony size. At all the inoculum sizes, microcolonies were observed after incubation with gentamicin at concentrations below 0.5 μg/ml; however, no colonies were observed when 1 μg/ml gentamicin was applied. We tested 17 clinically important antimicrobials with *E. coli* ATCC 25922 in the same range of inoculum sizes and compared the MIC values at each inoculum size (see Fig. 2B and Supplementary Fig. S4). There were 30 cases of a two-fold difference at all inoculum sizes and one case in which the MIC value showed two two-fold differences, at 5.0 × 10^5^ and 5.0 × 10^7^ CFU/ml, resulting in a 99.33% essential agreement rate (see Fig. 2C). We performed the same test by applying various antimicrobials to *P. aeruginosa* ATCC 27853, *S. aureus* ATCC 29213 and *E. faecalis* ATCC 29212, and the essential agreement rates were 100%, 95.37% and 100%, respectively. We concluded that inoculum size did not have a significant effect on the AST results in the dRAST system. The dRAST system was capable of detecting a wide range of bacterial concentrations with no difference in AST results, encompassing almost all PBCB concentrations with a dynamic range from 5 × 10^7^ to 5 × 10^9^ CFU/ml. It has been established that in AST, MICs tend to increase at inoculum sizes larger than the standard (5 × 10^5^ CFU/ml)^38–41^. However, the dRAST system was free of this phenomenon even at the high inoculum size of 5 × 10^9^ CFU/ml. In the dRAST system, the PBCB is diluted 100-fold with CAMHB and mixed with agarose at a 1:3 volume ratio; then, only 10 µl of the mixture is added to the well. Therefore, the final concentration of bacteria in the well is 1.25 × 10^6^ CFU/ml, only a two-fold difference from the standard inoculum. At the low inoculum size of 5 × 10^7^ CFU/ml, the final concentration of bacteria in the well is 1.25 × 10^4^ CFU/ml, which might make it difficult to detect bacterial growth. However, the dRAST system involves observations of microcolony formation from a single bacterium in agarose; this low limit of detection allows accurate MIC determination at low concentrations.

### Automatic AST using microcolony-forming area detection using image processing algorithm

**Figure 3:**
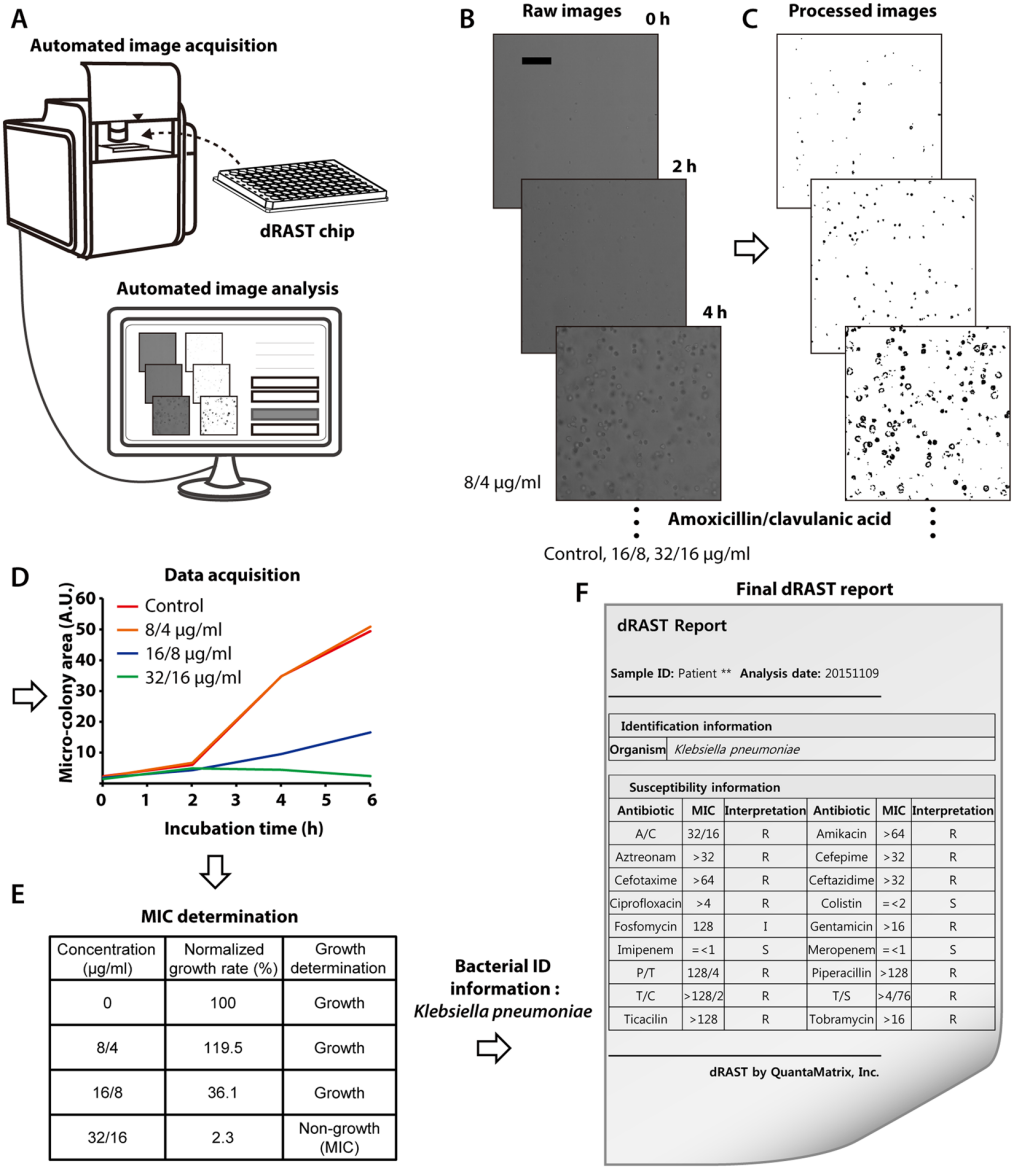
Automatic AST using colony-forming area detection. **(A)** Time-lapse images were acquired from the automated imaging system, and the image data were transferred to the analysis system. **(B)** Time-lapse images of *K. pneumoniae* with several concentrations of amoxicillin/clavulanic acid (A/C). **(C)** The raw images from **(B)** were processed to binary format images. The number of white pixels in the image represents the microcolony area. **(D)** Graph of the microcolony-forming area in **(C)**. From low A/C concentrations until 16/8 μg/ml, there was a substantial increase in microcolony-forming area in the images. However, from 16/8 μg/ml to 32/16 μg/ml, there was no substantial change in the area of bacteria. **(E)** The normalized growth rates at all the concentrations were calculated. Values higher than 20% were regarded as growth, and lower values were regarded as non-growth. From low A/C concentrations until 16/8 μg/ml, the normalized growth rates were higher than 20% and regarded as growth. However, at 32/16 μg/ml, the normalized growth rate was 2.3% and lower than 20%; thus, it was regarded as non-growth. Therefore, the MIC value was determined to be 32/16 μg/ml. **(F)** Final AST report. Using the ID information and MIC interpretive criteria from CLSI, antibiotic susceptibility was determined in a full antimicrobial panel. The scale bars in **(B)** represent 100 μm.

Clinical use of the dRAST system requires an automated system of time-lapse imaging through the final AST result. In our system, raw images were obtained from the prepared dRAST chip with an automated image acquisition system using a time-lapse method (see Fig. 3A). For example, a clinical strain, *K. pneumoniae*, was tested with amoxicillin/clavulanic acid (A/C) for the determination of MIC. Raw images were automatically analyzed to determine the MIC from serial dilution tests with antibiotics. The main role of this program is to calculate the area of colony formation in the images, which is accomplished by calculating the area occupied by bacteria. The raw images were transformed into a binary format through several processes (see Fig. 3B,C and Supplementary Fig. S3). After the area in the binary format image was calculated, a growth curve was generated (see Fig. 3D). The initial images sometimes contained blood cells or some debris. To compensate for this debris, the growth rate of the colony formation area was calculated as in eqn. 1.

1 $${A}_{S,N}=\,\frac{{A}_{S,F}-{A}_{S,I}}{{A}_{C,F}-{A}_{C,I}}$$

where *S* and *C* represent the sample and control, respectively; *F* and *I* represent the final and initial times of the image, respectively; and *A*_*S,N*_ represents the normalized growth rate of the area of a sample microcolony in the images. To determine MIC, a distinction between growth and non-growth is needed, which requires a threshold value. In broth microdilution (BMD) test, the MIC is the lowest concentration of antimicrobial agent that completely inhibits growth of the organism in microdilution wells as detected by the unaided eye^42^. However, the imaging method detected some growth at high concentrations that was not detected using the BMD method. We performed a spike-in test with four standard strains – *E. coli* ATCC 25922*, S. aureus* ATCC 29213, *P. aeruginosa* ATCC 27853, and *E. faecalis* ATCC 29212 – and compared the QC range from CLSI to establish a threshold value. Based on an inspection of all the images, we determined that 20% growth compared with the control well was considered ‘growth’. However, for several combinations of bacteria and antimicrobials, the threshold values were adjusted because the growth rate over six hours of incubation was relatively slow or fast. For example, for tetracycline with *S. aureus* ATCC 29213, the growth rate was decelerated due to the antimicrobial effect; therefore, the threshold value was reduced to allow agreement with the BMD result (see Supplementary Table S3 and Supplementary Fig. S8). Using this method, four standard strains were tested with clinically important antimicrobials in the dRAST chip, and the MIC values were calculated. To verify that the MIC values from dRAST were consistent with those from the gold standard method, the BMD test was performed simultaneously. In most cases, the MIC values were consistent with the values from BMD (see Supplementary Table S5). In an example case, the normalized growth rate was greater than 20% until 16/8 μg/ml and lower than 20% at 32/16 μg/ml, implying a MIC of 32/16 μg/ml (see Fig. 3E). For the final determination of antimicrobial susceptibility, bacterial identification (ID) information is needed because MIC value must be interpreted using MIC interpretive standards, which vary with bacterial species. Next, antibiotic susceptibility was determined in a full antimicrobial panel (see Fig. 3F).

### Direct and rapid AST integrated with bacterial ID using MALDI-TOF MS

**Figure 4:**
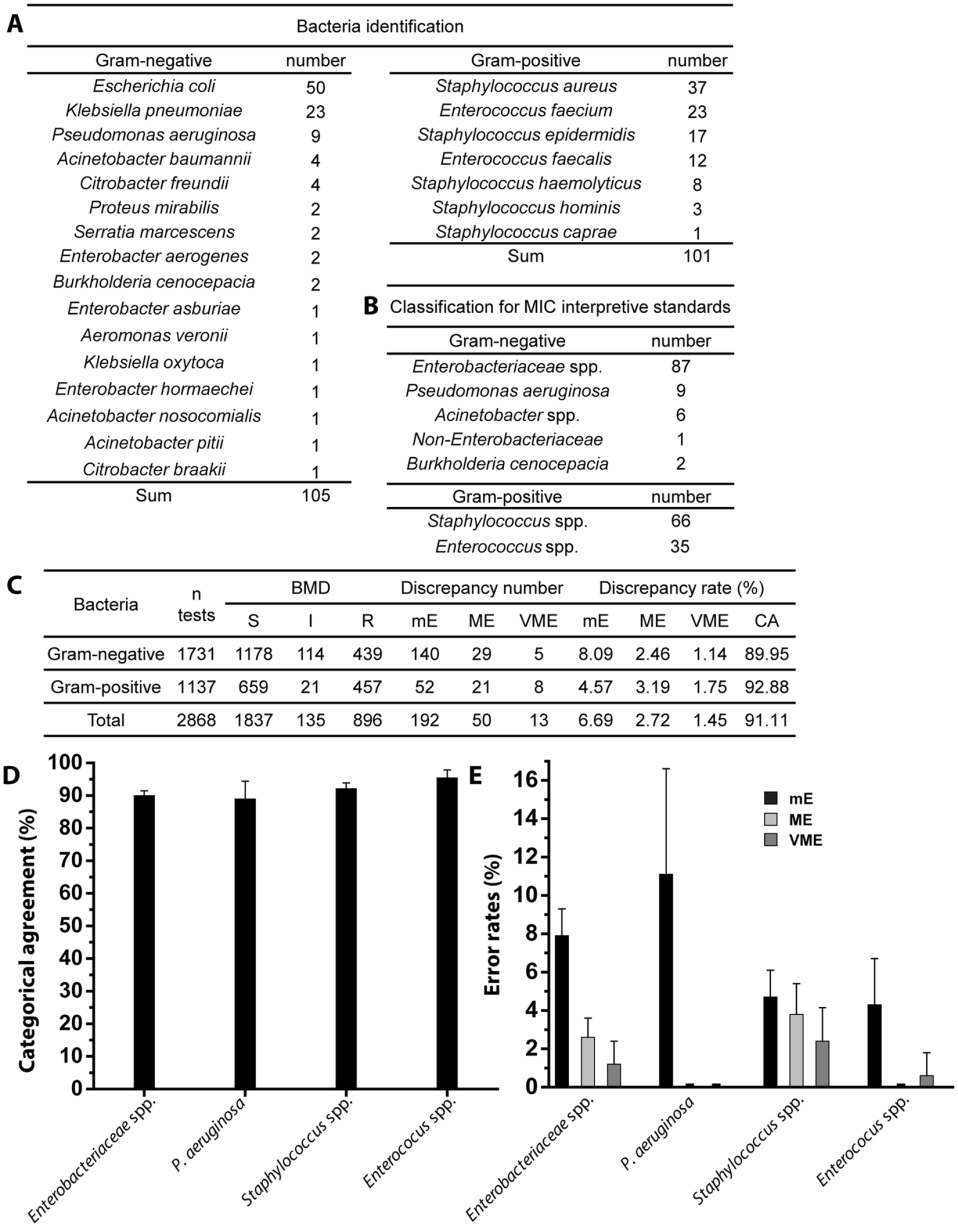
Summary of clinical testing with dRAST using colony-forming area detection. **(A)** Bacterial ID of 206 clinically isolated strains. There were 16 species of 105 Gram-negative strains and 7 species of 101 Gram-positive strains. **(B)** Classification of clinical samples for MIC interpretive standards. **(C)** Discrepancy rates and CA rates for dRAST using clinical samples. The dRAST results were compared with the BMD results to calculate the discrepancy rates. For the BMD test: S, susceptible; I, intermediate; R, resistant. For dRAST: mE, minor error; ME, major error; VME, very major error; CA, categorical agreement. **(D)** CA rates according to the main classified strains: *Enterobacteriaceae* spp. (n = 87, 89.9%), *P. aeruginosa* (n = 9, 88.9%), *Staphylococcus* spp. (n = 66, 92.1%), *Enterococcus* spp. (n = 35, 95.4%). **(E)** Discrepancy rates according to the main classified strains: *Enterobacteriaceae* spp. (mE = 7.9%, ME = 2.6%, VME = 1.2%), *P. aeruginosa* (mE = 11.1%, ME = 0.0%, VME = 0.0%), *Staphylococcus* spp. (mE = 4.7%, ME = 3.8%, VME = 2.4%), and *Enterococcus* spp. (mE = 4.3%, ME = 0.0%, VME = 0.6%). The error rates were calculated by comparing the AST results from each method with the BMD test. Error bars represent 95% confidence intervals.

We tested 105 Gram-negative and 101 Gram-positive strains from positive blood cultures (see Fig. 4A and B). After the dRAST test, the positive blood culture sample was inoculated onto an LB agar plate for subculture, and the CFU was measured. The bacterial concentration ranged from 1.3 × 10^8^ to 7.6 × 10^9^ CFU/ml in Gram-negative strains and 1.6 × 10^7^ to 7.6 × 10^9^ CFU/ml in Gram-positive strains (see Supplementary Fig. S5). Using the colony on agar plate, we performed the BMD test as a reference test to validate the performance of the dRAST system. The image processing program automatically derived the AST results from the dRAST images. The error rates were calculated by comparing the results from the dRAST system and BMD (see Fig. 4C). After 6 hours of incubation and image analysis, there was 11.6% of non-reliable growth in the sample that the image processing could not detect the substantial growth for determination of AST. 16 species of Gram-negative bacteria and seven species of Gram-positive bacteria were tested. Among the Gram-negative strains, 87 strains were classified as *Enterobacteriaceae* spp., and the others included nine *Pseudomonas aeruginosa* strains, six *Acinetobacter* spp. strains and three other species. Among the Gram-positive strains, 66 strains were classified as *Staphylococcus* spp., and 35 were classified as *Enterococcus* spp. These strains included strains of clinically important species, such as *Enterococcus faecium*, *Staphylococcus aureus*, *Klebsiella pneumoniae, Acinetobacter baumannii, Pseudomonas aeruginosa* and *Enterobacter* spp. (ESKAPE) strains. A total of 1731 tests were performed using Gram-negative strains, and there were 140 minor errors (mEs), 29 major errors (MEs) and 5 very major errors (VMEs) (see Supplementary Table S7). For determination of discrepancies, discrepancies are classified as follows according to FDA guidance. mE stands for which reference results is resistant or susceptible and device results is intermediate; reference result is intermediate and device result is resistant or susceptible. ME stands for which reference result is susceptible and device result is resistant. VME represents for which reference results is resistant and device result is susceptible. Among the 29 MEs, seven MEs occurred in trimethoprim/sulfamethoxazole (T/S). The 5 VMEs occurred in one in colistin, fosfomycin and tobramycin and two in piperacillin. The VMEs in Gram-negative strains included four from *Enterobacteriaceae* spp. with fosfomycin, piperacillin and tobramycin as well as one from *Acinetobacter* spp. with colistin (see Supplementary Tables S8–S10). For the Gram-positive strains, 1137 tests were conducted, and there were 52 mEs, 21 MEs and 8 VMEs (see Supplementary Table S11). Among the nine VMEs in Gram-positive strains, three VMEs were from penicillin and one was from oxacillin, erythromycin, imipenem and T/S in *Staphylococcus* spp. (see Supplementary Tables S12 and S13). There were 11 MEs in T/S with Gram-positive strains. In T/S, small microcolonies were observed even in susceptible cases, and the difference between susceptible and resistant was not clear. In some cases, the microcolonies in the processed images were out of focus and were not detected (see Supplementary Fig. S6). In cases in which the number of microcolonies was smaller than the number in the control, the area was not 20% larger than the control, and the sample was regarded as susceptible, i.e., possibly resistant in the BMD test. The categorical agreement rates for the dRAST clinical samples according to the main classified strains were 89.9% in *Enterobacteriaceae* spp., 88.9% in *P. aeruginosa*, 92.1% in *Staphylococcus* spp. and 95.4% in *Enterococcus* spp. (see Fig. 4D). The discrepancy rates are shown in Fig. 4E. The total number of *P. aeruginosa* samples in the clinical testing was nine, and most of the mEs were due to filament formation induced by beta-lactam antibiotics. Some cases were classified as ‘growth’ because the low-magnification imaging system was not capable of differentiating between division and filament formation. The results showed a categorical agreement of 91.11%, minor error of 6.69%, major error of 2.72%, and very major error of 1.45%. According to the US FDA, for the acceptable performance of susceptibility tests, the overall categorical agreement should be higher than 90%, with mEs ≤ 10%, MEs ≤ 3% and VMEs ≤ 1.5%^43^. Including all these reasons for error, the dRAST system satisfied the FDA’s recommended performance guidelines.

**Figure 5:**
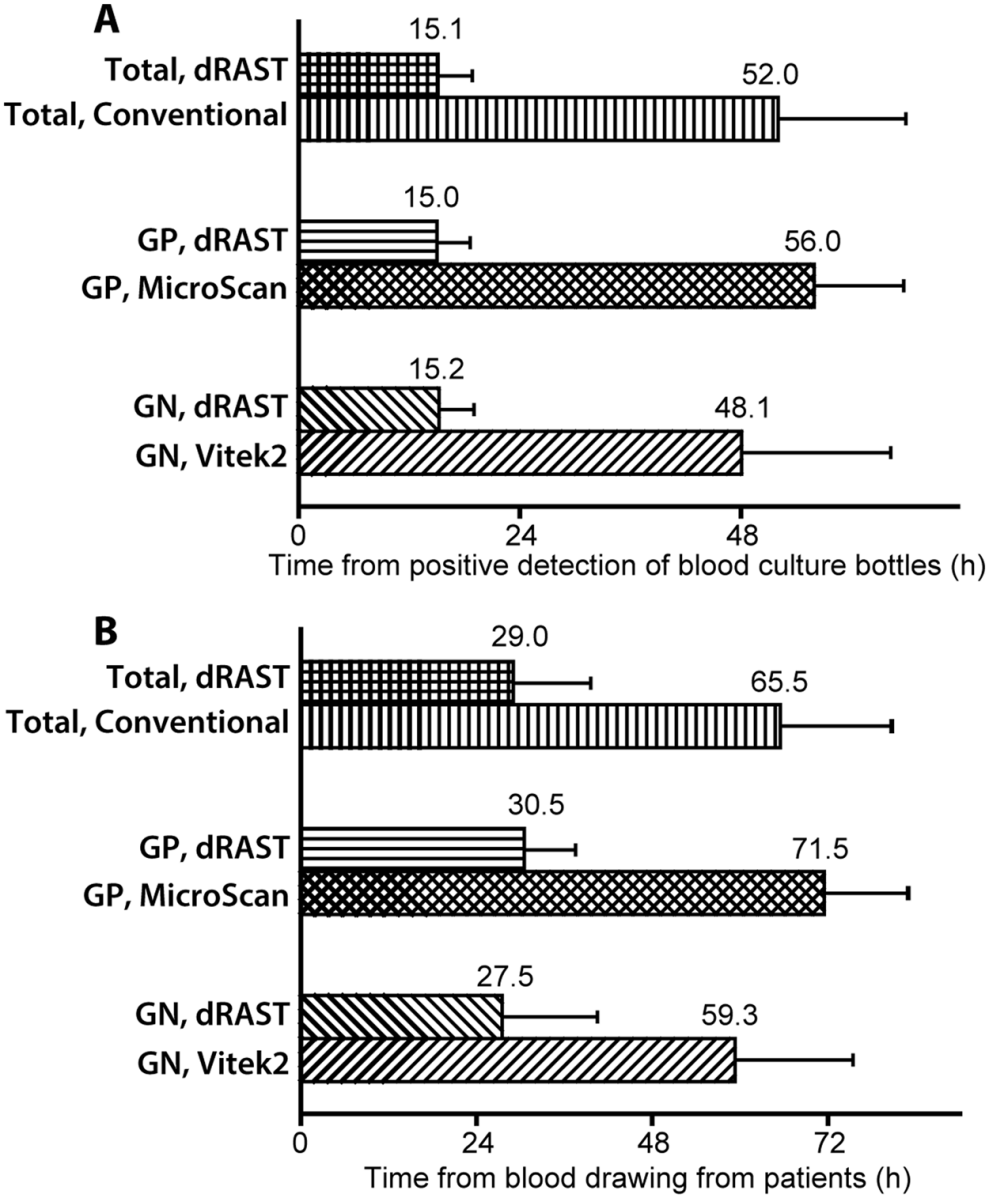
Average time to results of AST from (**A**) time of positive detection of blood culture bottles or (**B**) time of blood collection from patient. The time required for AST using dRAST and a conventional AST method requiring subculture (Vitek 2 or MicroScan) was calculated.

The time required for AST was compared using dRAST and the conventional method, Vitek 2 or MicroScan (see Fig. 5). In the case of dRAST, the average turnaround time for AST from a PBCB was 15.1 hours which includes the delay time in average nine hours between positive signal of blood culture and initiation of dRAST (see Fig. 5A). The time required for AST was only six hours; the remaining turnaround time was due to delayed tests flagged as positive. In the workflow of a clinical setting, flagged blood culture samples from overnight culture would be batch processed in the morning. The time required for AST using Vitek 2 and MicroScan, which include a subculture process, was 48.1 hours and 56.0 hours, respectively. The total time from blood collection was also compared (see Fig. 5B). In both cases, dRAST saved approximately 36 hours compared to the conventional methods.

## Discussion

Rapid and accurate antimicrobial prescriptions are critically needed by BSI patients. The current AST process, which is the test to determine the appropriate antimicrobial prescription, takes approximately three days from blood collection. To shorten this time, direct AST methods using PBCBs with no separation process could be an attractive candidate. However, performing direct AST from PBCBs is technically difficult because the inoculum size cannot be effectively controlled, substances in the specimens such as antimicrobial absorption materials in blood cultures could affect the results, and the bottle could contain a polymicrobial culture containing more than two organisms^44^. In conventional AST, which measures the optical density in a test well, the inoculum size must be controlled for consistent AST results. In the dRAST system, the response of individual bacteria to antimicrobials was measured by observing microcolony formation from a single bacterium. Therefore, this method can generate consistent AST results with a wide range of inoculum sizes, from the typical bacterial concentrations in PBCBs. Additionally, the PBCB aliquot is diluted 4000-fold in the final test well, and additional substances in the blood culture specimens did not affect the AST result. Therefore, the accuracy of AST result from the dRAST system satisfied the FDA’s recommendation.

When they are flagged as positive for growth by the blood culture instrument, cultured bacteria from patients are mixed with hemocytes: ~10^9^ erythrocytes and ~10^6^ leukocytes per 1 ml of blood. The conventional AST systems Vitek 2 and MicroScan are not capable of differentiating the change in optical density (OD) of culture media mixed with hemocytes. Therefore, these systems require a subculture process involving an additional 1 day of incubation. In this study, we developed a dRAST device using PBCBs without subculture process. We previously introduced a rapid AST method of imaging bacteria immobilized in an agarose gel^15, 16^. In the present work, we exploited the fact that bacterial culture media only allow bacterial cells to divide.

As mentioned above, polymicrobial infection is a fundamental barrier to direct AST. Approximately 5~10% of BSI cases are polymicrobial infections, and their mortality is higher than for monomicrobial infections^45, 46^. MALDI-TOF cannot identify multiple organisms in polymicrobial cultures^29^. A trial was conducted to evaluate the identification of polymicrobial using differences between the MS fingerprints of bacteria^47^. Using the dRAST system, we were able to detect polymicrobial infections in some cases involving substantial differences in microcolony size, shape and in the reaction to the antibiotics (see Supplementary Fig. S7). However, in other cases, the dRAST system could not detect polymicrobial infections. In addition to the limitation of current system, there were 11.6% of non-reliable growth cases in clinical study. Since dRAST system was not fully automated, imaging times were set to 0, 2, 4 and 6 hours after incubation and imaging after 6 hours for the non-reliable growth cases after examining the growth condition was not feasible due to the technical limitation. This issue should be handled in the fully automated system which control multiple dRAST chips and real time image processing to determine the AST results. Also, there is chance of different growth pattern after 6 hours of incubation in certain bacterial strains. The broad database on growth pattern according to the bacterial ID could contribute to predict the case and reduce the error.

Here, by eliminating the subculture process, the dRAST system could perform AST in six hours through microcolony detection under a microscope. Consequently, the dRAST system reduced the time from a positive signal from blood culture until AST from 40 hours to six hours. Assuming practical utilization in laboratory medicine, it would be possible to shorten the time required to obtain results by two days. The average time to detect a positive blood culture is approximately 16 hours^48^. Therefore, the total time from blood collection to the AST result was 24 hours using the dRAST system. Additionally, the dRAST system satisfied the performance recommendations of the US FDA. According to a literature survey, the time reduction in AST could decrease mortality and hospitalization periods^6–9^. The dRAST system could increase the survival rate of sepsis patients.

For the accurate prescription of antimicrobials to patients, ID information on the bacteria is a prerequisite for interpreting the MIC results from AST because the MIC interpretation criteria vary according to the species of bacteria^49^. Conventional AST systems, such as Vitek 2, MicroScan and Phoenix (Becton Dickinson Company, NJ, United States), perform the identification of bacteria simultaneously with AST. The dRAST system also requires an ID system for optimal antimicrobial treatment. To keep pace with the direct and rapid AST using PBCBs, the ID result should be obtained prior to the AST result. Recently, the MALDI-TOF MS system was validated for direct ID of bacterial species from positive blood cultures in one hour, including preparation time^29, 30^. Combining the dRAST system with direct ID using a MALDI Biotyper and Sepsityper kit from Bruker (Billerica, MA, United States) could create massive synergy in rapid BSI diagnosis. By eliminating the subculture process and using the PBCB directly in AST, we could receive the AST result 1 day sooner. Moreover, if the AST method could be performed in six hours, the AST result could be available within 24 hours after blood collection (see Fig. 1A). Combining direct ID using MALDI-TOF MS and dRAST will enable the ID and AST information to be provided on the same day as the detection of the bacteria from the blood. To achieve a walkaway AST system, we are developing a fully automated system that prepares the dRAST chip from a PBCB aliquot, performs time-lapse imaging of the plate every hour and determines the antimicrobial susceptibility.

For the time reduction of AST from blood collection, the blood culture step must be shortened. The dRAST system requires only 10^5^ CFU/ml of bacteria for AST, a value that is 10,000-fold smaller than the current bacterial concentration in positive blood culture bottles. A few trials have evaluated the rapid detection of bacteria in blood culture bottles^50^. Another method to shorten the duration of AST involves performing dRAST immediately after the positive detection of bacteria in the culture bottle. The dRAST system can perform direct and rapid AST from a PBCB with no separation process. The automation system integrating the blood culture and dRAST could reduce the time required for AST. If a new system capable of detecting positive blood cultures at an earlier stage is integrated with the dRAST system, the total time required for AST from blood collection would be 12 hours. This method could change the paradigm of antimicrobial prescription in the clinic and increase the survival rates of sepsis patients.

## Electronic supplementary material

Supplementary materials with changes marked

Data file S1
