# Supplementary material for: Direct, rapid antimicrobial susceptibility test from positive blood cultures based on microscopic imaging analysis
Source: Sci Rep. 2017 Apr 25;7:1148. doi: 10.1038/s41598-017-01278-2 (PMC5430693; doi:10.1038/s41598-017-01278-2)
Supplement: Supplementary file 1 — Supplementary materials with changes marked [file 41598_2017_1278_MOESM1_ESM.pdf]

Supplementary Information for

Direct, rapid antimicrobial susceptibility test from positive blood cultures based on microscopic imaging analysis

J. Choi[1]†, H. Y. Jeong[2,3]†, G. Y. Lee[2,4], S. Han[1], S. H. Han[1], B. Jin[1], T. Lim[1,2,4], S. Kim[5], D. Y. Kim[1], H. C. Kim[5,6,7], E.-C. Kim[8], S. H. Song[8], T. S. Kim[8], S. Kwon[1,2,3,4,9]*

*To whom correspondence should be addressed: E-mail: skwon@snu.ac.kr (S.K.)

†These authors contributed equally to this article.

This PDF file includes:

Supplementary Methods

Figures

Fig. S1. Number of bacteria in PBCBs.

Fig. S2. Time-lapse images of microcolonies from different dilution factors.

Fig. S3. Image processing for microcolony detection.

Fig. S4. Consistent AST results from a wide range of inoculum sizes.

Fig. S5. Number of bacteria in clinical PBCB samples.

Fig. S6. Time-lapse images of microcolonies of *Staphylococcus hominis* with trimethoprim/sulfamethoxazole.

Fig. S7. Detection of polymicrobial infection using (A) Size difference. Filled triangles represent P. aeruginosa and unfilled triangles represent E. coli after six hours of incubation. (B) Shape difference.

Fig. S8. Time-lapse detection of decelerated growth rate due to antimicrobial effect

Tables

Table S1. Performance of MALDI-TOF/Sepsityper for identification of bacteria from positive blood culture bottles.

Table S2. Information of non-reliable growth in clinical sample test.

Table S3. Optimized threshold values.

Table S4. The heating process during the freeze-drying process.

Table S5. MIC values from dRAST with spiked sample, broth microdilution test and CLSI quality control ranges.

Table S6. Number of bacteria in the well and field of view (FOV).

Table S7. Summary of clinical testing of Gram-negative strains using dRAST, according to the antimicrobial applied.

Table S8. Summary of clinical testing of *Enterobacteriaceae* spp. strains using dRAST, according to the applied the antimicrobial applied.

Table S9. Summary of clinical testing of *Pseudomonas aeruginosa* strains using dRAST, according to the antimicrobial applied.

Table S10. Summary of clinical testing of *Acinetobacter* spp. strains using dRAST, according to the antimicrobial applied.

Table S11. Summary of clinical testing of Gram-positive strains using dRAST, according to the antimicrobial applied.

Table S12. Summary of clinical testing of *Staphylococcus* spp. strains using dRAST according to the applied antimicrobial applied.

Table S13. Summary of clinical testing of *Enterococcus* spp. strains using dRAST according to the antimicrobial applied.

Other Supplementary Material for this manuscript includes the following:

Data file S1. Positive blood culture bottles information.

Supplementary Methods

Image acquisition

For the detection of bacteria, magnification higher than 400x is necessary. However, in this system, a bacterial microcolony is imaged rather than a single bacterium. Therefore, relatively low 100x magnification imaging (a 20x lens with a 5x tube lens or a 10x lens with a 10x tube lens) was used. The 96-well format dRAST chip was scanned, and each well in the chip was imaged in a custom-made automated imaging system. The imaging system consisted of an xy-axis motor (Ezi-Step-42S, Fastech, Inc., Republic of Korea) with a motion controller (DMC-B140, Galli Motion Control, Rocklin, CA, United States), a z-axis motor (SAN4505-150PR+3SR, Sciencetown, Inc., Republic of Korea) with a motion controller (PMC-1HS, Autonics Sensors & Controllers, Republic of Korea), a CMOS camera (Ximea, USB3 Vision xiQ MQ022CG-CM, Münster, Germany), a 20x objective lens (Olympus, UPlanFL N, Tokyo, Japan), and related electronics. The automated operation of the system was controlled using in-house-developed PC software. Predefined wells in the chip, divided based on antibiotic panel type (Gram-positive and Gram-negative), were scanned automatically to obtain images of microcolonies. To find an imaging area in each well and an identical imaging plane in the z-axis across all wells with different vertical profiles, we used a focus mark injection-molded as a half-cross shape on the bottom of each well. The image was processed to find the position and contrast of the focus mark while vertically scanning the well bottom. The z-axis reference position that produced the greatest contrast of the focus mark image was obtained by vertically scanning the well bottom (first coarse scan range/step: 468.75/46.87 μm, second fine scan range/step: 112.5/5.62 μm). At the same time, the xy reference position was obtained from the position of the mark in the image found by pattern-matching of the half-cross shape. Finally, the microcolony image was acquired after moving the xyz stage by the amount of the predefined offsets.

Colony formation monitoring

Colony formation in the dRAST chip was monitored by a QMAP (QuantaMatrix microscopic analysis platform) automated image analyzer with a total magnification of 100x using a 20x lens

and a 5x tube lens. The imaging area was 1126.4 μm x 594 μm. Time-lapse images were acquired in the same area.

Image processing

For the automated determination of antimicrobial susceptibility, we developed automated image processing software. An automated image analysis program was written using C++ (Microsoft Visual Studio 2013) and OpenCV (Version 3.0, http://opencv.org) and was integrated with the imaging system program for real-time image processing. The image analysis process is composed of 3 steps: 1) sharpening to emphasize the microcolony, 2) binarization to detect the microcolony, and 3) object counting and sizing to calculate the growth of the microcolony (Fig. S3). Sharpening was performed by subtracting an image blurred using a Gaussian filter from its own un-blurred image; the image blurring was achieved by applying a Gaussian filter with a window size of 5 by 5 pixels, and different weights were given to the original image and blurred image (1.5 and 0.5, respectively) for subtracting the two images. Then, adaptive thresholding was applied for the binarization to obtain different thresholds for different regions of the same image to compensate for differences in illumination in the image. The threshold value was determined as the weighted sum of neighborhood pixel values, where weights were a Gaussian window with a size of 49 by 49 pixels. From the binary image, microcolonies were detected by finding the contours of objects composed of pixels over the binarization thresholds after dilating 2 times to close the contours. Finally, the detected contours were counted and sized to calculate the growth of the microcolonies.

Antimicrobial preparations

The antimicrobial agents were purchased from Sigma-Aldrich (MO, United States) and Santa Cruz Biotechnology, Inc. (CA, United States). Stock solutions were prepared using the method recommended by the supplier and the CLSI guidelines[43]. The stock solutions were stored at -70 °C. In the case of ceftazidime, the antimicrobial solutions were prepared directly from powdered form following the manufacturer's instructions.

Freeze-dried antimicrobials

The antimicrobials were freeze-dried in the dRAST chip and broth microdilution plates for immediate testing. The antimicrobial was prepared at a 10-fold higher concentration than the actual concentrations to be tested. Then, 10 μl of concentrated antimicrobial solution was loaded into the satellite well in the dRAST chip. For lyophilization, we used a PVTFD 10R programmable vacuum freeze dryer (Ilshin Lab, Kyunggi-Do, Republic of Korea). The freeze-drying process involved a freezing tray, freezing trap, vacuum and heating tray. The heating process during the freeze-drying process is shown in Table S4. After freeze-drying process, the dRAST chip was stored at 4 °C with desiccating agent.

Reference AST: broth microdilution (BMD) test

For the reference AST, we performed BMD test using freeze-dried antibiotic solutions in 96-well microtiter plate (BD Biosciences, CA, United States) as dRAST chip. A 100 μl of CAMHB was added to the freeze-dried antibiotics for the appropriate concentration, which was determined by the CLSI recommendation[43]. 10 μl of bacterial stock solution was inoculated into the well at a final concentration of 5 x 10^5 CFU/ml. The BMD tests were performed in triplicate. After 16–20 hours of incubation at 37 °C, the MIC value of each microdilution well was determined by comparing the amount of growth in the wells containing the antimicrobial agent with the amount of growth in the growth-control wells by unaided visual inspection. Totally reduction of growth is determined as non-growth. With trimethoprim and the sulfonamides, the MIC value of each microdilution well was determined as the concentration at which there was ≥80% reduction in growth compared with the control. If the results from the triplicate tests were not identical, the majority of result was selected as the MIC.

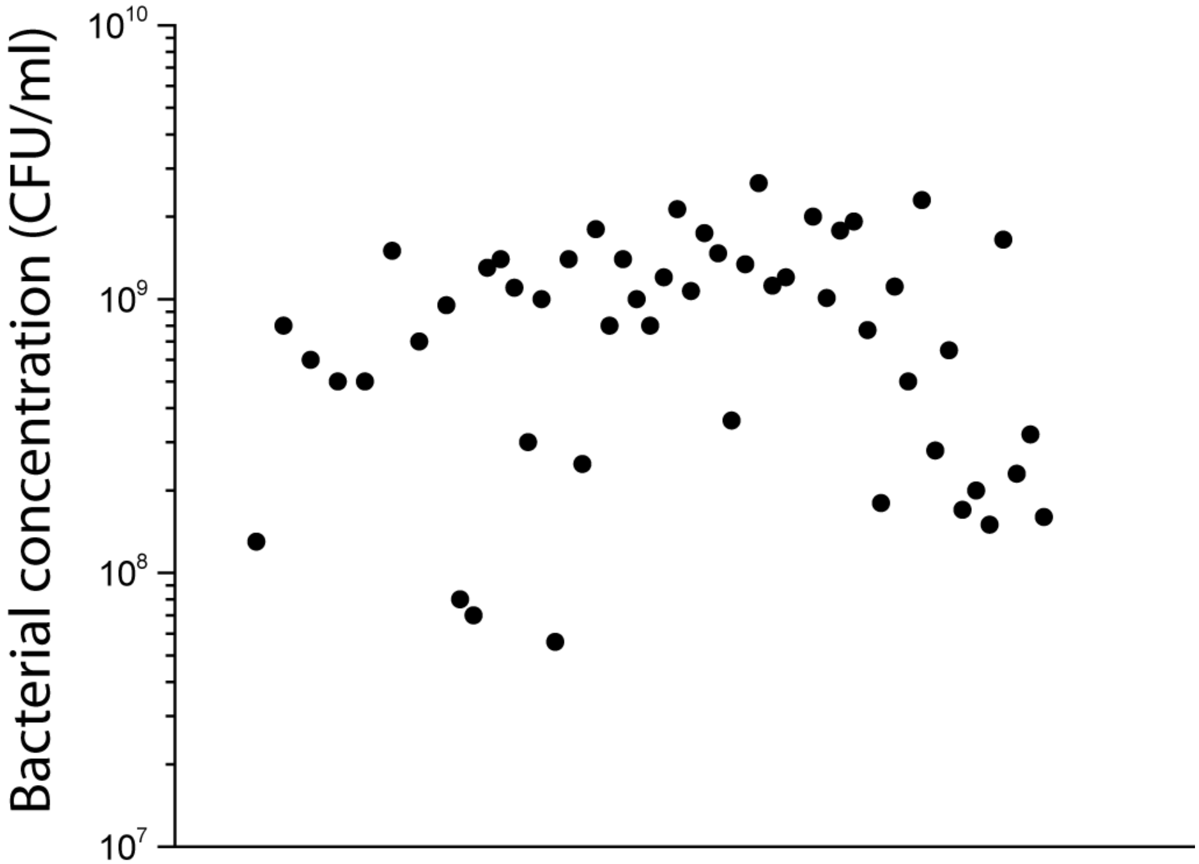

Fig. S1. Number of bacteria in PBCBs. The average number of bacteria in PBCBs was approximately 9.4 x 10^8 CFU/ml. The highest concentration was approximately 2.5 x 10^9 CFU/ml, and the lowest was approximately 5.6 x 10^7 CFU/ml.

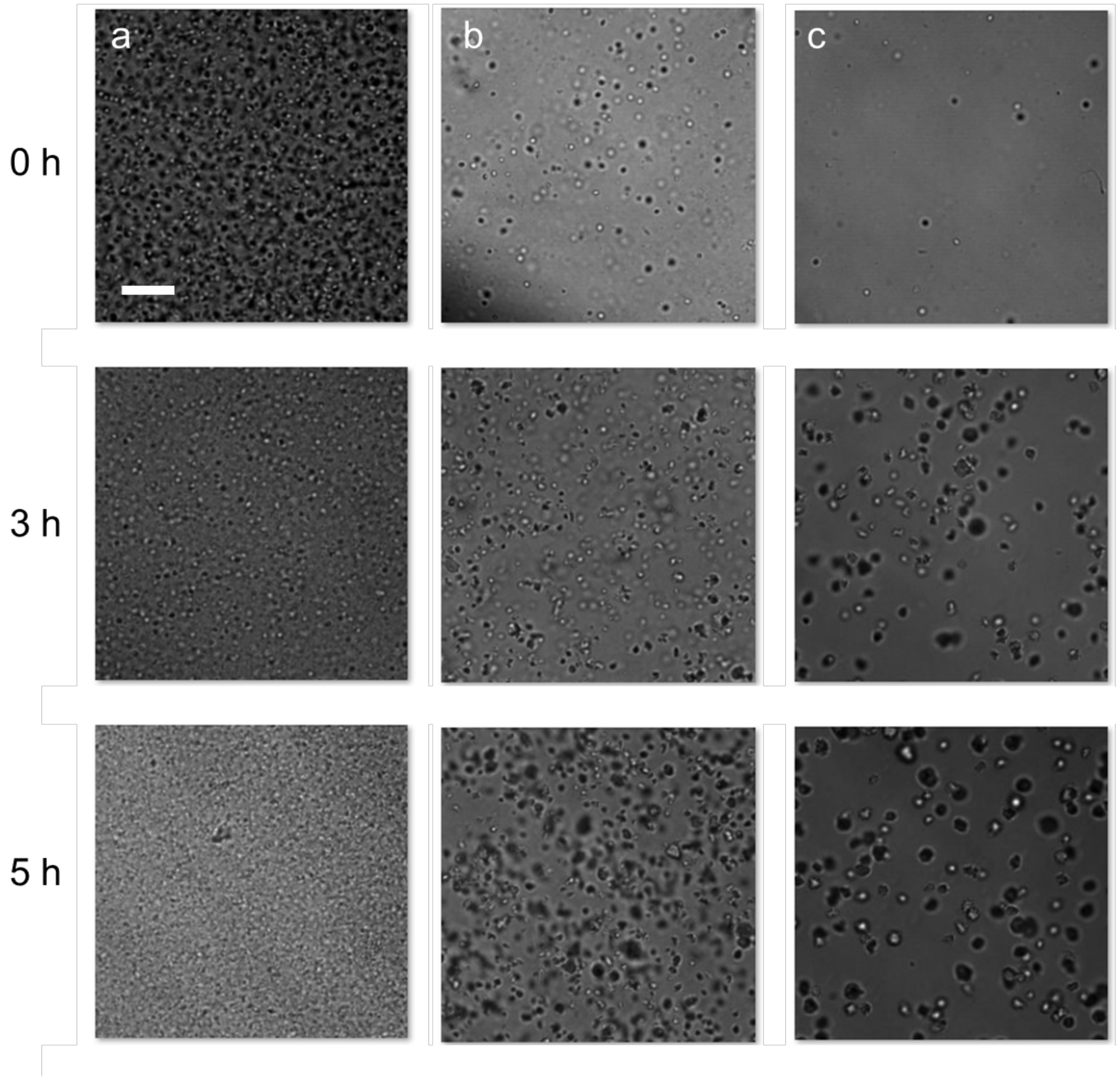

Fig. S2. Time-lapse images of microcolonies from different dilution factors. (a) The raw sample from the PBCB was mixed with agarose. (b) A 10x dilution sample from PBCB. (c) A 100x dilution sample from PBCB. The scale bar represents 100 μm.

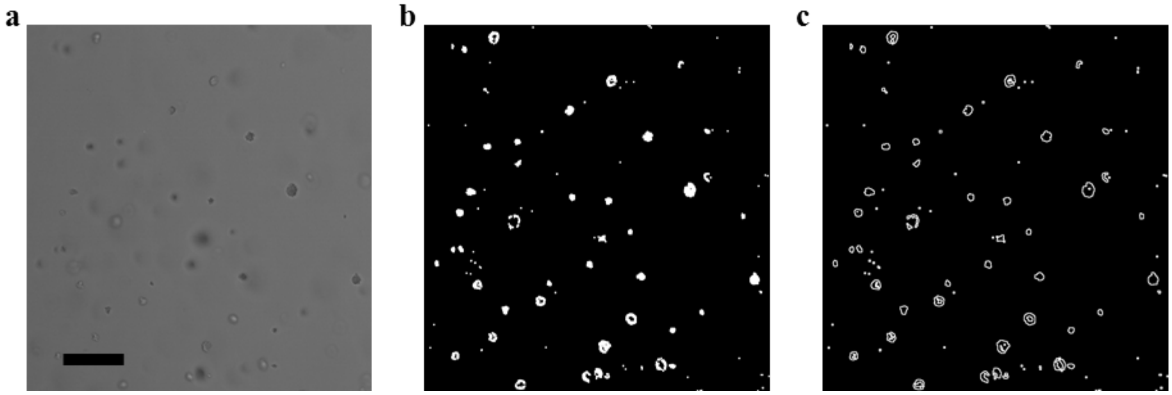

Fig. S3. Image processing for microcolony detection. (a) The raw image acquired from the imaging system. (b) The result of sharpening and binarization. (c) The detected contours of the microcolonies recognized in (b). The scale bar represents 300 μm.

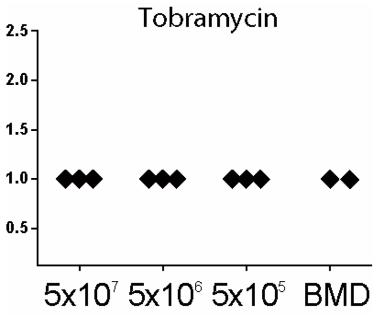

Fig. S4. Consistent AST results from a wide range of inoculum sizes. MIC values from different *E. coli* ATCC 25922 inoculum sizes of 5.0 x 10^7, 5.0 x 10^6 and 5.0 x 10^5 CFU/ml incubated with tobramycin and analyzed using dRAST and broth microdilution test.

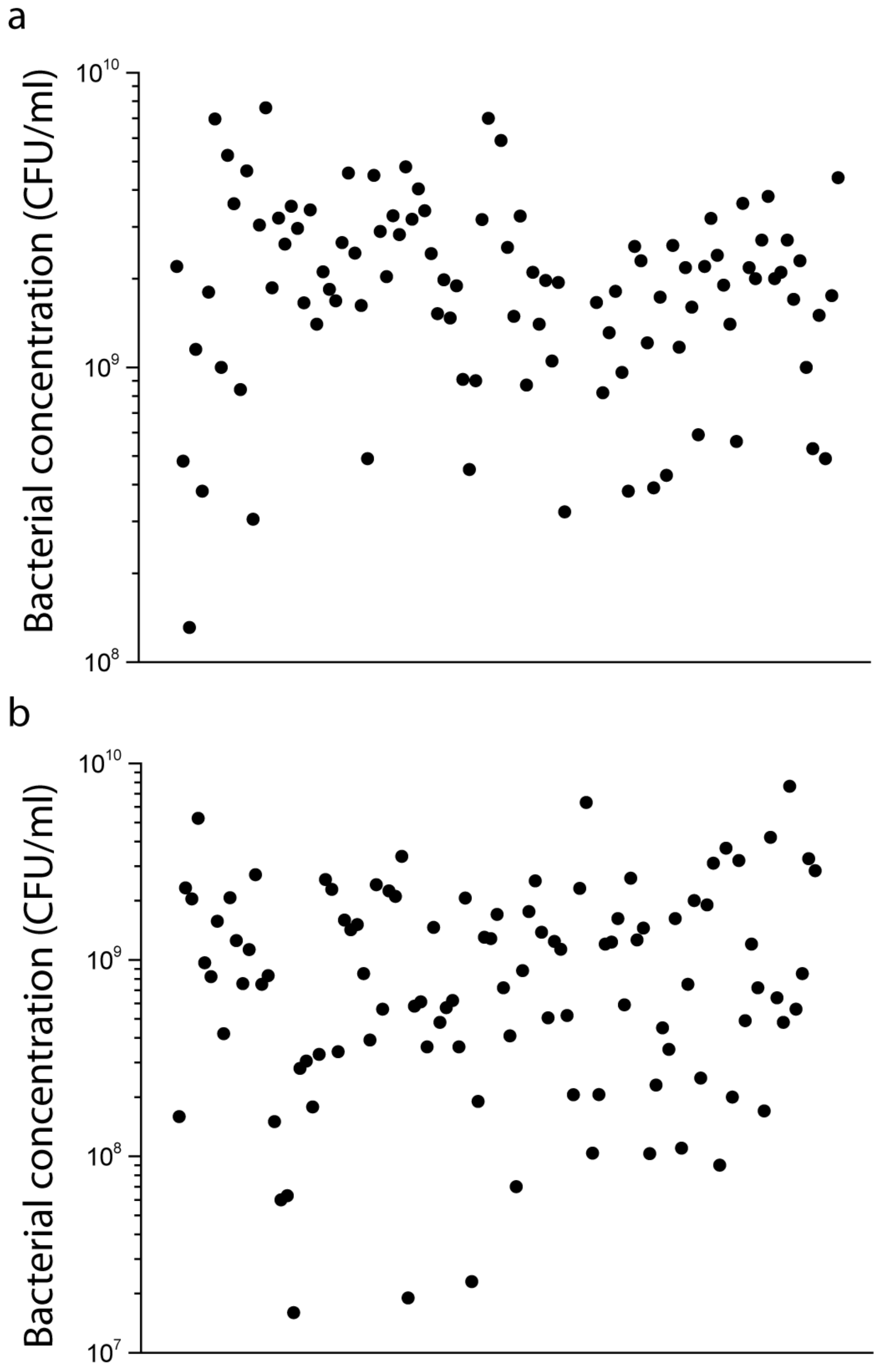

Fig. S5. Number of bacteria in clinical PBCB samples. (a) Gram-negative strain bottle. The highest concentration was approximately 7.6 x 10^9 CFU/ml, and the lowest was approximately 1.3 x 10^8 CFU/ml. (b) Gram-positive strain bottle. The highest concentration was approximately 7.6 x 10^9 CFU/ml, and the lowest was approximately 1.6 x 10^7 CFU/ml.

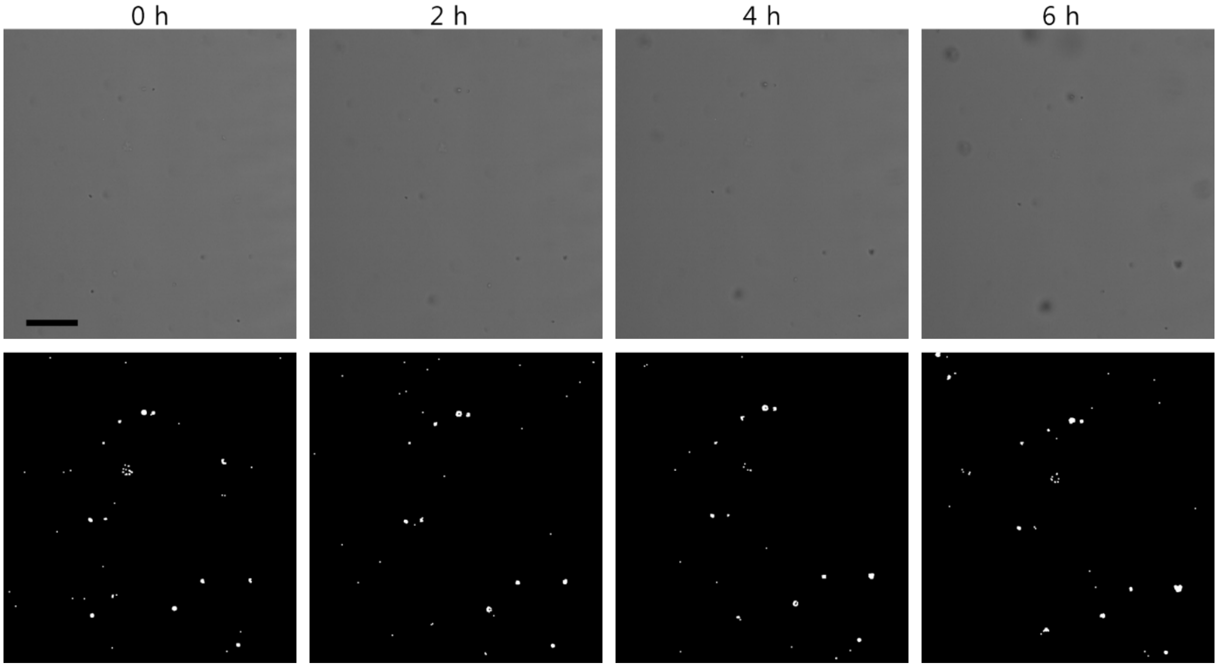

Fig. S6. Time-lapse images of microcolonies of *Staphylococcus hominis* with trimethoprim/sulfamethoxazole. In the raw images, few microcolonies were visible. However, some of the microcolonies were out of focus and not detected in the processed images. The scale bar represents 300 µm.

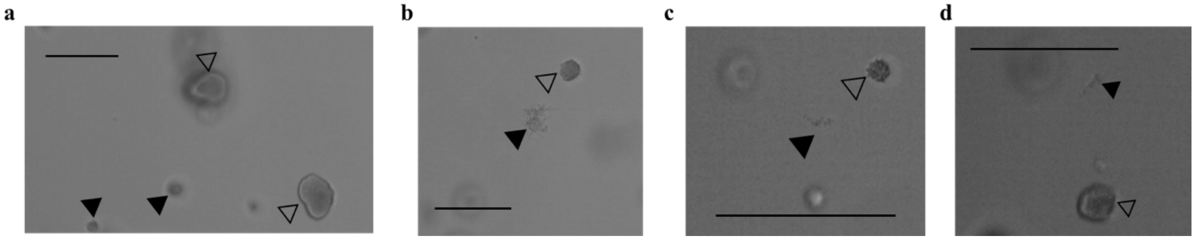

Fig. S7. Detection of polymicrobial infection using (A) Size difference. Filled triangles represent *P. aeruginosa* and unfilled triangles represent *E. coli* after six hours of incubation. (B) Shape difference. Filled triangle represents *P. urinalis* and unfilled triangle represents *S. aureus* after six hours of incubation. (C) Different reactions to the antibiotic aztreonam. Filled triangle represents filament formation of *P. aeruginosa* and unfilled triangle represents *S. aureus* after four hours of incubation. (D) Different reactions to the antibiotic ceftazidime. Filled triangle represents filament formation of *P. aeruginosa* and unfilled triangle represents *S. aureus* after six hours of incubation. All scale bars represent 100 µm.

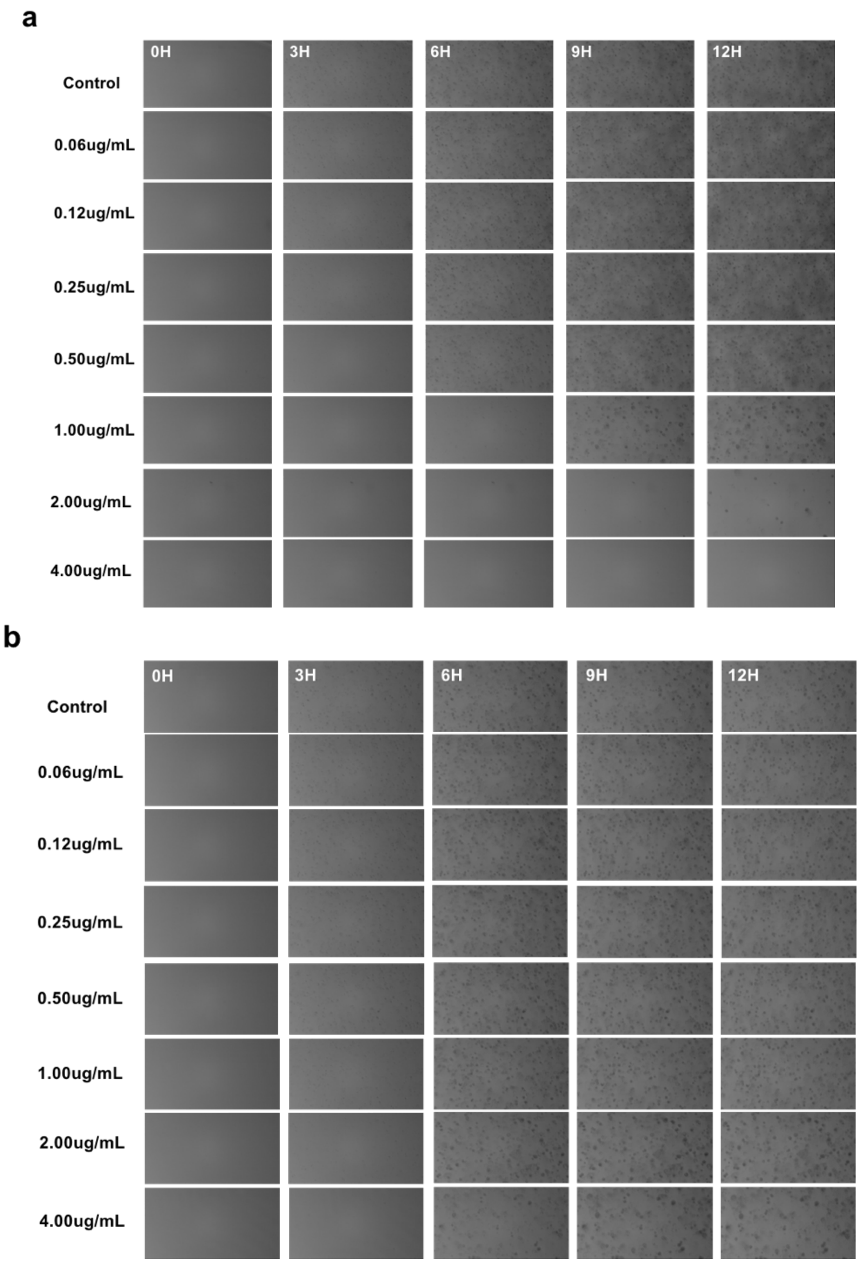

Fig. S8. Time-lapse detection of decelerated growth rate due to antimicrobial effect

(A) Time-lapse detection of *S. aureus* to the antibiotic erythromycin. (B) Time-lapse detection of *E. faecalis* to the antibiotic erythromycin.

Table S1. Performance of MALDI-TOF/Sepsityper for identification of bacteria from positive blood culture bottles. (a) Summary of performance of MALDI-TOF/Sepsityper for identification of bacteria from positive blood culture bottles. (b) Total cases of disagreement between MALDI-TOF/Sepsityper identification and conventional method (Vitek 2 and MicroScan). (c) Polymicrobial cases that there was only one species in MALDI-TOF/Sepsityper and there were two kinds of colonies in subculture plate and ID from conventional method.

(a)

	**Number of samples**	**Agreement rate (%)**
Total number of sample	206	-
Agreement in genus level	205	99.5
Agreement in species level	197	95.6

(b)

**ID from MALDI-TOF MS**	**MALDI-TOF MS score**	**ID from conventional method (Method)**	**Discrepancy level**
Enterobacter hormaechei	1.989	*Enterobacter cloacae* (Vitek2)	Species
Enterobacter asburiae	2.024	*Enterobacter cloacae* (Vitek2)	Species
Aeromonas veronii	1.931	*Aeromonas hydrophila* (Vitek2)	Species
Staphylococcus caprae	1.769	*Staphylococcus epidermidis* (MicroScan)	Species
Staphylococcus epidermidis	1.587	*Staphylococcus aureus* (MicroScan)	Species
Enterobacter aerogenes	1.92	*Klebsiella pneumoniae* (Vitek2)	Genus
Staphylococcus aureus	2.289	*Staphylococcus*, Coagulase negative (MicroScan)	Species
Acinetobacter pitii	1.868	*Acinetobacter baumannii* (Vitek2)	Species
Acinetobacter nosocomialis	1.807	*Acinetobacter baumannii* (Vitek2)	Species

(c)

**ID from MALDI-TOF MS**	**MALDI-TOF MS score**	**ID from conventional method (Method)**	**Discrepancy level**
Escherichia coli	2.0	*Escherichia coli, Aeromonas hydrophila* (Vitek2)	-
Escherichia coli	2.151	*Escherichia coli, Aeromonas hydrophila* (Vitek2)	-

Table S2. Information of non-reliable growth in clinical sample test.

Bacterial Identification	Number of non-reliable growth samples
Staphylococcus aureus	7
Staphylococcus epidermidis	4
Klebsiella pneumoniae	3
Pseudomonas aeruginosa	3
Staphylococcus hominis	3
Enterobacter aerogenes	2
Escherichia coli	2
Acinetobacter pittii	1
Aeromonas veronii	1
Citrobacter koseri	1
Total	27
Percentage of non-reliable growth	11.6%

Table S3. Optimized threshold values. The general threshold value to determine the MIC was 0.2. The threshold values were optimized in the following cases.

**Threshold values of *E. coli* ATCC 25922 for *Enterobacteriaceae* spp. and other Gram-negative species**		**Threshold values of *P. aeruginosa* ATCC 27853 for *P. aeruginosa***	
**Antimicrobial**	**Threshold value**	**Antimicrobial**	**Threshold value**
Ciprofloxacin	0.42	Imipenem	0.26
Amikacin	0.355	Cefotaxime	0.26
Imipenem	0.5	Fosfomycin	0.29
Meropenem	0.37	Ticarcillin/ clavulanic acid	0.43
		Aztreonam	0.6
		Piperacillin	0.26
		Ciprofloxacin	0.34
		Gentamicin	0.35
		Ticarcillin	0.67

**Threshold values of *S. aureus* ATCC 29213 for *Staphylococcus* spp.**		**Threshold values of *E. faecalis* ATCC 29212 for *Enterococcus* spp.**	
**Antimicrobial**	**Threshold value**	**Antimicrobial**	**Threshold value**
Ampicillin	0.15	Gentamicin	0.09
Oxacillin	0.32	Tetracycline	0.13
Tetracycline	0.113		
Levofloxacin	0.13		
Clindamycin	0.14		
Imipenem	0.29		

Table S4. The heating process during the freeze-drying process. After freezing tray, freezing trap and vacuum process in freeze-drying, the tray was heated for decrease the water content in antibiotics. The heating process was composed of several sequences.

	Sequence #1	Sequence #2	Sequence #3	Sequence #4	Sequence #5	Sequence #6
Temp (°C)	-40	-20	-10	0	10	20
Vac (mTorr)	0	0	0	0	0	0
Set Time (Min)	60	60	60	60	60	∞

Table S5. MIC values from dRAST with spiked sample, broth microdilution test and CLSI quality control ranges. The dRAST test was performed on a positive blood culture sample spiked with four standard strains. The broth microdilution test was performed on a colony cultured on an LB agar plate. Each test was performed in three times.

Antimicrobial	MIC, dRAST (μg/ml)	MIC, BMD (μg/ml)	CLSI QC range (μg/ml)	MIC, dRAST (μg/ml)	MIC, BMD (μg/ml)	CLSI QC range (μg/ml)
	**(A) Gram-negative strains**					
	E. coli ATCC 25922			*P. aeruginosa* ATCC 27853		
Amikacin	2,4	2,4	0.5-4	2	1-4	1-4
Amoxicillin/ clavulanic acid	8/4	8/4	2/1-8/4	-	-	-
Aztreonam	0.25	0.12-0.5	0.06-0.25	4	2,4	2-8
Cefepime	0.06, 0.12	0.03, 0.12	0.015-0.12	2	1,2	0.5-4
Cefotaxime	-	-	-	32	16	8-32
Ceftazidime	-	-	-	2,4	2	1-4
Ciprofloxacin	-	-	-	0.25,0.5	0.25,0.5	0.25-1
Colistin	2	0.5,1	0.25-2	2,4	1	0.5-4
Fosfomycin	-	-	-	2,4	8	2-8
Gentamicin	1	0.25-1	0.25-1	1,2	1,2	0.5-2
Imipenem	-	-	-	2,4	4	1-4
Meropenem	-	-	-	0.5,1	0.5	0.25-1
Piperacillin	4	4	1-4	4,8	4	1-8
Piperacillin/ tazobactam	4/4	2/4,4/4	1/4-4/4	8/4	8/4	1/4-8/4
Trimethoprim/ sulfamethoxazole	≤0.5/9.5	≤0.5/9.5	≤0.5/9.5	-	-	-
Ticarcillin	16	8,16	4-16	16,32	16,32	8-32
Ticarcillin/ clavulanic acid	4/2	8/2, 16/2	4/2-16/2	-	-	-
Tobramycin	1	0.5,1	0.25-1	0.5,1	0.25,0.5	0.25-1
	**(B) Gram-positive strains**					
	S. aureus ATCC 29213			*E. faecalis* ATCC 29212		
Ampicillin	1	1,2	0.5-2	2	2	0.5-2
Ciprofloxacin	0.12,0.25	0.25,0.5	0.12-0.5	2	2	0.25-2
Clindamycin	0.12,0.25	0.12, 0.25	0.06-0.25	8,16	8, 16	4-16
Erythromycin	0.25, 0.5	0.5	0.25-1	2	1, 2	1-4
Gentamicin	0.5	0.25, 0.5	0.12-1	4	8	4-16
Imipenem	-	-	-	1,2	2	0.5-2
Levofloxacin	0.25	0.25	0.06-0.5	0.5	0.5, 1	0.25-2
Linezolid	1	4	1-4	1,2	2	1-4
Oxacillin	0.5	0.25, 0.5	0.12-0.5	16	8,16	8-32
Penicillin	0.5	0.5,1	0.25-2	2	2	1-4
Rifampin	-	-	-	1	1,2	0.5-4
Tetracycline	0.25,0.5	0.5,1	0.12-1	8	16,32	8-32

Trimethoprim/ sulfamethoxazole	≤0.5/9.5	≤0.5/9.5	≤0.5/9.5	≤0.5/9.5	≤0.5/9.5	≤0.5/9.5
Vancomycin	1	1,2	0.5-2	2,4	4	1-4

Table S6. Number of bacteria in the well and field of view (FOV). We diluted the PBCBs by 1/100. On average, the final concentration was 9.4×10^6 CFU/ml. After mixing with agarose at a 1:3 volume ratio, the concentration was 2.3×10^6 CFU/ml. In this case, the final concentration in the well was 2×10^4 CFU, and there were approximately 100 bacteria cells in the field of view of the 20X lens with a 5x tube lens.

	Number of bacteria (CFU)				
	1 ml of positive blood culture bottle	1 ml of 100x dilution	1 ml of mixture with agarose	10 µl of mixture in the well	In FOV of 20x lens
Average	9.4×10^8	9.4×10^6	2.3×10^6	2.3×10^4	160
Min	5.6×10^7	5.6×10^5	1.4×10^5	1.4×10^3	10
Max	2.6×10^9	2.6×10^7	6.5×10^6	6.5×10^4	460

Table S7. Summary of clinical testing of Gram-negative strains using dRAST, according to the antimicrobial applied.

Antimicrobial	n tests	BMD			Discrepancy number			Discrepancy rate (%)			
		S	I	R	mE	ME	VME	mE	ME	VME	CA
Amoxicillin/clavulanic acid	90	52	18	20	18	1	0	20.00%	1.92%	0.00%	78.89%
Amikacin	105	98	0	7	0	0	0	0.00%	0.00%	0.00%	100.00%
Aztreonam	99	64	7	28	6	6	0	6.06%	9.38%	0.00%	87.88%
Cefepime	105	71	5	29	11	1	0	10.48%	1.41%	0.00%	88.57%
Cefotaxime	96	59	2	35	2	5	0	2.08%	8.47%	0.00%	92.71%
Ceftazidime	105	70	2	33	10	2	0	9.52%	2.86%	0.00%	88.57%
Ciprofloxacin	105	63	5	37	5	0	0	4.76%	0.00%	0.00%	95.24%
Colistin	15	13	1	1	3	0	1	20.00%	0.00%	100.00%	73.33%
Fosfomycin	87	49	11	27	19	0	1	21.84%	0.00%	3.70%	77.01%
Gentamicin	105	81	0	24	0	0	0	0.00%	0.00%	0.00%	100.00%
Imipenem	105	86	5	14	8	0	0	7.62%	0.00%	0.00%	92.38%
Meropenem	105	92	1	12	1	0	0	0.95%	0.00%	0.00%	99.05%
Piperacillin/tazobactam	105	88	2	15	6	4	0	5.71%	4.55%	0.00%	90.48%
Piperacillin	102	52	4	46	17	2	2	16.67%	3.85%	4.35%	79.41%
Ticarcillin/clavulanic acid	102	51	23	28	17	0	0	16.67%	0.00%	0.00%	83.33%
Trimethoprim/ Sulfamethoxazole	96	70	0	26	0	7	0	0.00%	10.00%	0.00%	92.71%
Ticarcillin	102	41	22	39	11	0	0	10.78%	0.00%	0.00%	89.22%
Tobramycin	102	78	6	18	6	1	1	5.88%	1.28%	5.56%	92.16%
Total	1731	1178	114	439	140	29	5	8.09%	2.46%	1.14%	89.95%

Table S8. Summary of clinical testing of *Enterobacteriaceae* spp. strains using dRAST, according to the applied the antimicrobial applied.

Antimicrobial	n tests	BMD			Discrepancy number			Discrepancy rate (%)			
		S	I	R	mE	ME	VME	mE	ME	VME	CA
Amoxicillin/clavulanic acid	87	50	18	19	17	1	0	19.54%	2.00%	0.00%	79.31%
Amikacin	87	84	0	3	0	0	0	0.00%	0.00%	0.00%	100.00%
Aztreonam	87	59	3	25	3	6	0	3.45%	10.17%	0.00%	89.66%
Cefepime	87	62	3	22	10	1	0	11.49%	1.61%	0.00%	87.36%
Cefotaxime	87	56	1	30	1	5	0	1.15%	8.93%	0.00%	93.10%
Ceftazidime	87	61	2	24	9	2	0	10.34%	3.28%	0.00%	87.36%
Ciprofloxacin	87	54	4	29	4	0	0	4.60%	0.00%	0.00%	95.40%
Colistin	-	-	-	-	-	-	-	-	-	-	-
Fosfomycin	87	49	11	27	19	0	1	21.84%	0.00%	3.70%	77.01%
Gentamicin	87	69	0	18	0	0	0	0.00%	0.00%	0.00%	100.00%
Imipenem	87	80	3	4	6	0	0	6.90%	0.00%	0.00%	93.10%
Meropenem	87	85	0	2	0	0	0	0.00%	0.00%	0.00%	100.00%
Piperacillin/tazobactam	87	79	1	7	5	4	0	5.75%	5.06%	0.00%	89.66%
Piperacillin	87	46	4	37	15	2	2	17.24%	4.35%	5.41%	78.16%
Ticarcillin/clavulanic acid	87	49	19	19	14	0	0	16.09%	0.00%	0.00%	83.91%
Trimethoprim/ Sulfamethoxazole	87	66	0	21	0	6	0	0.00%	9.09%	0.00%	93.10%
Ticarcillin	87	40	17	30	8	0	0	9.20%	0.00%	0.00%	90.80%
Tobramycin	87	69	6	12	6	1	1	6.90%	1.45%	8.33%	90.80%
Total	1479	1058	92	329	117	28	4	7.91%	2.65%	1.22%	89.93%

Table S9. Summary of clinical testing of *Pseudomonas aeruginosa* strains using dRAST, according to the antimicrobial applied.

Antimicrobial	n tests	BMD			Discrepancy number			Discrepancy rate (%)			
		S	I	R	mE	ME	VME	mE	ME	VME	CA
Amoxicillin/clavulanic acid	-	-	-	-	-	-	-	-	-	-	-
Amikacin	9	9	0	0	0	0	0	0.00%	0.00%	nan%	100.00%
Aztreonam	9	2	4	3	2	0	0	22.22%	0.00%	0.00%	77.78%
Cefepime	9	5	2	2	1	0	0	11.11%	0.00%	0.00%	88.89%
Cefotaxime	-	-	-	-	-	-	-	-	-	-	-
Ceftazidime	9	5	0	4	0	0	0	0.00%	0.00%	0.00%	100.00%
Ciprofloxacin	9	6	0	3	0	0	0	0.00%	0.00%	0.00%	100.00%
Colistin	9	8	1	0	3	0	0	33.33%	0.00%	nan%	66.67%
Fosfomycin	-	-	-	-	-	-	-	-	-	-	-
Gentamicin	9	7	0	2	0	0	0	0.00%	0.00%	0.00%	100.00%
Imipenem	9	2	2	5	2	0	0	22.22%	0.00%	0.00%	77.78%
Meropenem	9	3	1	5	1	0	0	11.11%	0.00%	0.00%	88.89%
Piperacillin/tazobactam	9	5	1	3	0	0	0	0.00%	0.00%	0.00%	100.00%
Piperacillin	9	5	0	4	1	0	0	11.11%	0.00%	0.00%	88.89%
Ticarcillin/clavulanic acid	9	1	4	4	2	0	0	22.22%	0.00%	0.00%	77.78%
Trimethoprim/ Sulfamethoxazole	-	-	-	-	-	-	-	-	-	-	-
Ticarcillin	9	0	5	4	2	0	0	22.22%	-	0.00%	77.78%
Tobramycin	9	7	0	2	0	0	0	0.00%	0.00%	0.00%	100.00%
Total	126	65	20	41	14	0	0	11.11%	0.00%	0.00%	88.89%

Table S10. Summary of clinical testing of *Acinetobacter* spp. strains using dRAST, according to the antimicrobial applied.

Antimicrobial	n tests	BMD			Discrepancy number			Discrepancy rate (%)			
		S	I	R	mE	ME	VME	mE	ME	VME	CA
Amoxicillin/clavulanic acid	-	-	-	-	-	-	-	-	-	-	-
Amikacin	6	2	0	4	0	0	0	0.00%	0.00%	0.00%	100.00%
Aztreonam	-	-	-	-	-	-	-	-	-	-	-
Cefepime	6	1	0	5	0	0	0	0.00%	0.00%	0.00%	100.00%
Cefotaxime	6	0	1	5	1	0	0	16.67%	-	0.00%	83.33%
Ceftazidime	6	1	0	5	1	0	0	16.67%	0.00%	0.00%	83.33%
Ciprofloxacin	6	1	0	5	0	0	0	0.00%	0.00%	0.00%	100.00%
Colistin	6	5	0	1	0	0	1	0.00%	0.00%	100.00%	83.33%
Fosfomycin	-	-	-	-	-	-	-	-	-	-	-
Gentamicin	6	2	0	4	0	0	0	0.00%	0.00%	0.00%	100.00%
Imipenem	6	1	0	5	0	0	0	0.00%	0.00%	0.00%	100.00%
Meropenem	6	1	0	5	0	0	0	0.00%	0.00%	0.00%	100.00%
Piperacillin/tazobactam	6	1	0	5	1	0	0	16.67%	0.00%	0.00%	83.33%
Piperacillin	6	1	0	5	1	0	0	16.67%	0.00%	0.00%	83.33%
Ticarcillin/clavulanic acid	6	1	0	5	1	0	0	16.67%	0.00%	0.00%	83.33%
Trimethoprim/ Sulfamethoxazole	6	1	0	5	0	1	0	0.00%	100.00%	0.00%	83.33%
Ticarcillin	6	1	0	5	1	0	0	16.67%	0.00%	0.00%	83.33%
Tobramycin	6	2	0	4	0	0	0	0.00%	0.00%	0.00%	100.00%
Total	90	21	1	68	6	1	1	6.67%	4.76%	1.47%	91.11%

Table S11. Summary of clinical testing of Gram-positive strains using dRAST, according to the antimicrobial applied.

Antimicrobial	n tests	BMD			Discrepancy number			Discrepancy rate			
		S	I	R	mE	ME	VME	mE	ME	VME	CA
Ampicillin	35	13	0	22	0	0	0	0.00%	0.00%	0.00%	100.00%
Ciprofloxacin	101	37	0	64	6	0	0	5.94%	0.00%	0.00%	94.06%
Clindamycin	66	51	1	14	1	0	0	1.52%	0.00%	0.00%	98.48%
Erythromycin	101	34	6	61	18	1	1	17.82%	2.94%	1.64%	80.20%
Gentamicin	66	37	6	23	5	0	0	7.58%	0.00%	0.00%	92.42%
Imipenem	66	41	0	25	0	4	1	0.00%	9.76%	4.00%	92.42%
Levofloxacin	101	37	0	64	3	0	0	2.97%	0.00%	0.00%	97.03%
Linezolid	101	100	1	0	2	2	0	1.98%	2.00%	nan%	96.04%
Oxacillin	65	28	0	37	0	0	1	0.00%	0.00%	2.70%	98.46%
Penicillin	101	14	0	87	0	1	3	0.00%	7.14%	3.45%	96.04%
Rifampin	66	52	2	12	2	1	0	3.03%	1.92%	0.00%	95.45%
Trimethoprim/ Sulfamethoxazole	66	52	0	14	0	11	1	0.00%	21.15%	7.14%	81.82%
Tetracycline	101	81	1	19	7	1	0	6.93%	1.23%	0.00%	92.08%
Vancomycin	101	82	4	15	8	0	1	7.92%	0.00%	6.67%	91.09%
Total	1137	659	21	457	52	21	8	4.57%	3.19%	1.75%	92.88%

Table S12. Summary of clinical testing of *Staphylococcus* spp. strains using dRAST according to the applied antimicrobial applied.

Antimicrobial	n tests	BMD			Discrepancy number			Discrepancy rate (%)			
		S	I	R	mE	ME	VME	mE	ME	VME	CA
Ampicillin	-	-	-	-	-	-	-	-	-	-	-
Ciprofloxacin	66	33	0	33	4	0	0	6.06%	0.00%	0.00%	93.94%
Clindamycin	66	51	1	14	1	0	0	1.52%	0.00%	0.00%	98.48%
Erythromycin	66	30	4	32	13	1	1	19.70%	3.33%	3.12%	77.27%
Gentamicin	66	37	6	23	5	0	0	7.58%	0.00%	0.00%	92.42%
Imipenem	66	41	0	25	0	4	1	0.00%	9.76%	4.00%	92.42%
Levofloxacin	66	33	0	33	2	0	0	3.03%	0.00%	0.00%	96.97%
Linezolid	66	66	0	0	0	2	0	0.00%	3.03%	-	96.97%
Oxacillin	65	28	0	37	0	0	1	0.00%	0.00%	2.70%	98.46%
Penicillin	66	4	0	62	0	1	3	0.00%	25.00%	4.84%	93.94%
Rifampin	66	52	2	12	2	1	0	3.03%	1.92%	0.00%	95.45%
Trimethoprim/ Sulfamethoxazole	66	52	0	14	0	11	1	0.00%	21.15%	7.14%	81.82%
Tetracycline	66	57	1	8	7	1	0	10.61%	1.75%	0.00%	87.88%
Vancomycin	66	62	4	0	6	0	0	9.09%	0.00%	-	90.91%
Total	857	546	18	293	40	21	7	4.67%	3.85%	2.39%	92.07%

Table S13. Summary of clinical testing of *Enterococcus* spp. strains using dRAST according to the antimicrobial applied.

Antimicrobial	n tests	BMD			Discrepancy number			Discrepancy rate (%)			
		S	I	R	mE	ME	VME	mE	ME	VME	CA
Ampicillin	35	13	0	22	0	0	0	0.00%	0.00%	0.00%	100.00%
Ciprofloxacin	35	4	0	31	2	0	0	5.71%	0.00%	0.00%	94.29%
Clindamycin	-	-	-	-	-	-	-	-	-	-	-
Erythromycin	35	4	2	29	5	0	0	14.29%	0.00%	0.00%	85.71%
Gentamicin	-	-	-	-	-	-	-	-	-	-	-
Imipenem	-	-	-	-	-	-	-	-	-	-	-
Levofloxacin	35	4	0	31	1	0	0	2.86%	0.00%	0.00%	97.14%
Linezolid	35	34	1	0	2	0	0	5.71%	0.00%	nan%	94.29%
Oxacillin	-	-	-	-	-	-	-	-	-	-	-
Penicillin	35	10	0	25	0	0	0	0.00%	0.00%	0.00%	100.00%
Rifampin	-	-	-	-	-	-	-	-	-	-	-
Trimethoprim/ Sulfamethoxazole	-	-	-	-	-	-	-	-	-	-	-
Tetracycline	35	24	0	11	0	0	0	0.00%	0.00%	0.00%	100.00%
Vancomycin	35	20	0	15	2	0	1	5.71%	0.00%	6.67%	91.43%
Total	280	113	3	164	12	0	1	4.29%	0.00%	0.61%	95.36%
